# A comprehensive benchmark of active learning strategies with AutoML for small-sample regression in materials science

**DOI:** 10.1038/s41598-025-24613-4

**Published:** 2025-10-23

**Authors:** Jinghou Bi, Yuanhao Xu, Felix Conrad, Hajo Wiemer, Steffen Ihlenfeldt

**Affiliations:** Faculty of Mechanical Science and Engineering, Dresden University of Technology DE, Dresden, 01069 Germany

**Keywords:** Engineering, Materials science, Mathematics and computing

## Abstract

The high cost and difficulty of acquiring labeled data in materials science often limits the scale of data-driven modeling efforts. Experimental synthesis and characterization often require expert knowledge, expensive equipment, and time-consuming procedures, making it critical to develop data-efficient learning strategies. Integrating Automated Machine Learning (AutoML) with active learning enables the construction of robust material-property prediction models while substantially reducing the volume of labeled data required. This benchmark study aims to evaluate various active learning (AL) strategies within AutoML in materials science regression tasks. The performance of each strategy in terms of model accuracy and data efficiency is analyzed. The 9 datasets used are derived from materials formulation design, which are typically small due to high data acquisition costs. 17 active-learning strategies, together with a Random-Sampling baseline, are systematically evaluated and compared for their effectiveness. Early in the acquisition process, uncertainty-driven (LCMD, Tree-based-R) and diversity-hybrid (RD-GS) strategies clearly outperform geometry-only heuristics (GSx, EGAL) and baseline, selecting more informative samples and improving model accuracy. As the labeled set grows, the gap narrows and all 17 methods converge, indicating diminishing returns from AL under AutoML.

## Introduction

In materials science, machine learning (ML) and deep learning (DL) methods applied to ever-growing tabular repositories are redefining how we screen, rank and ultimately create new compounds. Recent work on 50 000 low-symmetry perovskites demonstrated that gradient-boosting and support-vector regressors can compress the mean absolute error of band gap prediction to 0.18 eV, whereas SHAP analysis revealed the valence-charge variance as a decisive design lever^1^. Multi-objective optimisation frameworks now couple thermodynamic simulations with XGBoost to navigate millions of candidate chemistries; one such study downselected 2.5 million compositions to a single L1$_2$-strengthened single-crystal high-entropy alloy whose 800 °C yield strength reached 873 MPa^2^. Closing the loop between computation and synthesis, the autonomous “A-Lab” platform leveraged literature-mined recipes, first-principles phase-stability data and active learning to synthesize 41 previously unreported inorganic compounds within 17 days^3^. Collectively, these advances illustrate how ML-centric analysis of structured data is evolving from heuristic exploration to quantitatively reliable prediction, positioning data-driven models as an indispensable “second microscope” for accelerating material discovery.

A critical limitation of ML or DL methods, however, lies in their dependence on large labeled datasets for reliable performance. Model accuracy and generalizability are inherently tied to the availability of diverse, high-quality training data—a requirement that becomes prohibitively expensive when labeling demands domain expertise, specialized instrumentation, or intricate experimental protocols. This challenge is particularly acute in materials science, where the synthesis and characterization of labeled samples entail substantial costs and technical complexity^4^. For example, acquiring mechanical strength or thermal conductivity data for concrete composites requires meticulous synthesis, precise control of environmental conditions, and advanced analytical instrumentation^5^. Furthermore, inherent material variability often mandates repeated measurements to ensure statistical reliability, exacerbating both the time requirements and resource constraints. These barriers underscore the urgent need for data-efficient methodologies capable of building robust predictive models with minimal labeled samples.

To address data scarcity in materials science, researchers have pursued two complementary strategies: data-centric and model-centric approaches. The model-centric approach focuses on improving the learning algorithm itself to maintain robust predictive performance despite limited data conditions. In this line of thinking, researchers often refine the model structure, hyperparameter settings, and training schemes to improve the model’s adaptability and generalization ability. In recent years, the rapid development of Automated Machine Learning (AutoML) has further highlighted the potential of model-centric approaches: AutoML is capable of automatically searching and optimizing between different model families (e.g., tree models, neural networks) and their corresponding hyperparameters (e.g., learning rates, regularization coefficients), and even data preprocessing methods, which greatly reduces the user’s repetitive work in model design and parameterization ^6,7^. AutoML is particularly valuable in materials science, where experimentation and characterization are often time- and resource-intensive, making large-scale manual tuning impractical for practical applications. Meanwhile, AutoML has been proven to be an excellent tool for material design.^8,9^

Data-centric approaches such as augmentation^10^, synthetic-data generation (e.g., SMOTE, GANs)^11^, and transfer learning^12^ enrich training corpora ex situ; by contrast, AL directly modulates data acquisition itself and has therefore emerged as a particularly effective means of reducing annotation cost. In iterative AL cycles, query strategies based on uncertainty, diversity, or their hybrids dynamically select the most informative yet unlabeled candidates, thereby maximizing model performance under stringent data budgets. This property is especially valuable in materials discovery, where each new data point may require high-throughput computation or costly synthesis. For example, Lookman *et al.* showed that uncertainty-driven AL curtailed experimental campaigns in alloy design by more than 60%^13^, while Koizumi *et al.* achieved state-of-the-art accuracy for ternary phase-diagram regression using only 30% of the data typically required^14^. In addition to these studies, Li *et al.* systematically quantified redundancy in several million-entry first-principles databases and demonstrated that a query-by-committee AL scheme can reach both in-distribution and out-of-distribution performance parity with full-data baselines while querying merely  30% of the pool-equivalent to a 70–95% savings in computational or labeling resources; for certain band gap predictions, as little as 10% of the data were sufficient^15^. Collectively, these studies underscore that AL is not merely a convenient heuristic but also a quantitatively validated route to data-efficient, scalable materials informatics.

Despite these advances, critical gaps persist. First, AL’s effectiveness varies markedly across tasks, depending on data dimensionality, distribution, and initial sampling strategies^16^. Current studies often validate AL protocols on narrow datasets, risking overfitting and limiting generalizability^17^. Furthermore, while AutoML holds potential as a surrogate model for AL, no systematic benchmarks exist to guide strategy selection in multi-step AutoML workflow. Establishing a rigorous evaluation framework-employing diverse and dimensionally heterogeneous materials datasets under standardized evaluation protocols-is therefore imperative. Such a benchmark would not only quantify the robustness of AL strategies on the basis of the AutolML framework in complex regression scenarios, but also provide actionable further insights for optimizing experimental design and accelerating materials innovation.

Despite these advances, critical gaps persist. First, AL’s efficacy still varies markedly across tasks, depending on data dimensionality, distribution, and initial sampling strategies^16^. Second and most importantly, when AL is embedded in an AutoML pipeline the surrogate model is no longer static: the optimiser may switch-across iterations-from linear regressors to tree-based ensembles and eventually to neural networks, following whichever model family offers the optimal bias–variance–cost trade-off^6^. Consequently, an AL strategy must remain robust under dynamic changes in hypothesis space and uncertainty calibration, a requirement seldom considered in conventional AL studies that assume a fixed learner. However, to date, no systematic benchmark has quantified how well existing AL strategies cope with such model drift in multi-step AutoML workflows. Establishing a rigorous evaluation framework that spans dimensionally heterogeneous materials datasets under standardized protocols is therefore imperative. Such a benchmark will (i) reveal which sampling principles remain reliable when the underlying model family evolves, (ii) provide actionable guidance for selecting AL strategies inside AutoML, and ultimately (iii) accelerate materials discovery through more efficient, data-sparse experimentation.

## Methods

**Fig. 1:**
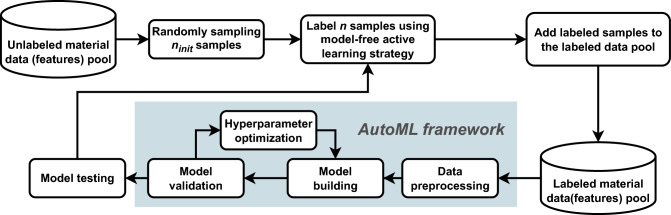
Dynamic data modeling pipeline for material design via AL integrated with AutoML. The highlighted steps indicate automated procedures within the AutoML workflow.

In the pool-based AL framework, a regression task scenario is addressed using an AutoML approach. The data processing pipeline is illustrated in Fig. 1. The initial dataset comprises a small set of labeled samples and a large pool of unlabeled samples. The labeled dataset $L = \{(x_i, y_i)\}_{i=1}^l$ contains $l$ samples, where $x_i \in \mathbb {R}^d$ is a $d$-dimensional feature vector, and $y_i \in \mathbb {R}$ is the corresponding continuous target value. The unlabeled data pool $U = \{x_i\}_{i=l+1}^n$ contains the remaining feature vectors. Active learning iteratively selects the most informative sample $x^*$ from $U$, which is expected to improve the model’s performance. The target value $y^*$ of the selected sample is then obtained through human annotation. The newly labeled sample is added to the labeled dataset, expanding the training set as follows: $L = L \cup \{(x^*, y^*)\}$ and the model is updated accordingly. This process continues until a stopping criterion is met. In benchmark experiments, the stopping criterion is typically defined as reaching a point where no additional samples can be obtained.

The specific benchmarking process is shown in Fig. 1. In this setting, an unlabeled dataset is assumed, and the objective is to select data samples of optimal value to the data-driven model (AutoML in this study) from the unlabeled dataset through AL, followed by labeling.

Specifically, $n_{init}$ samples are first randomly sampled from the unlabeled dataset as the initial labeled dataset. Afterwards, different AL strategies are used to perform multi-step sampling and put the sampled samples into the labeled dataset. An AutoML model is fitted in each sampling step and the model performance is tested.

In this work, the training and test sets are partitioned in 80:20 ratio and the validation of the model is performed automatically in the AutoML workflow. And the validation method is cross-validation with the number of folds set to 5.

The compared strategies include various principles based on predictive uncertainty, reducing model error, optimizing predictive variance, maximizing potential information gain, and hybrid variants of these methods. The testing process involves iterative sampling in multiple rounds, progressively expanding the labeled dataset and updating the regression model’s performance in real-time.

Model performance is evaluated using **Mean absolute error **(*MAE*) and **Coefficient of Determination **($R^2$), with each strategy’s effectiveness compared with random sampling. In the later stages, different strategies may gradually converge in performance, making their effectiveness during the early data-scarce phase particularly crucial. This benchmark includes a comparison of various AL methods, systematically evaluating their different characteristics in small-scale data scenarios and as the data volume increases, thereby providing a structured assessment for AL in regression tasks.

### Active learning strategies

In this benchmark test, multiple AL strategies evaluated are all based on four different principles: Uncertainty Estimation, Expected Model Change Maximization (EMCM), Diversity, and Representativeness. Each strategy is either based on a single principle or a combination of multiple principles.

Uncertainty estimation is one of the earliest studied principles of AL strategies. For classification tasks, uncertainty estimation is relatively easy to implement, with the most commonly used approach being vote entropy-based methods ^18,19^. However, implementing uncertainty estimation for regression tasks is more challenging, as there is no direct method to measure uncertainty in regression tasks. Currently, most uncertainty estimation methods are based on Monte Carlo dropout ^20,21^ and other variance reduction-based approaches ^22,23^. For example, **Monte Carlo Dropout** is a technique used to estimate the uncertainty of model predictions. During training, dropout serves as a regularization method by randomly deactivating neurons to prevent overfitting. In the testing phase, Monte Carlo Dropout keeps the dropout active, performing multiple forward passes on the same input to produce a distribution of outputs. The mean of these outputs is taken as the model’s prediction, while the variance indicates the uncertainty of that prediction.

Uncertainty-based query criteria were among the first ideas explored in pool-based AL and remain the dominant paradigm today. In classification, the model itself already returns a probability distribution over discrete labels, so measures such as vote entropy, margin sampling, or least-confidence can be computed directly and at virtually no extra cost^18,19^. In regression, the predictor outputs only a single continuous value, so the learner must first wrap the model in a probabilistic layer that yields both a mean and a variance before any uncertainty-driven acquisition rule can be applied. The prevailing practice is therefore to approximate Bayesian inference: Monte-Carlo Dropout keeps dropout active at test time, performs multiple stochastic forward passes on the same input, and uses the sample mean as the point prediction while interpreting the sample variance as epistemic uncertainty^20,21^. Alternative approximations include deep ensembles, whose variance across independently trained networks serves as the uncertainty score^24^; variance-reduction committee methods such as Query-by-Committee^22^; and fully Bayesian models such as Gaussian-process regression or Bayesian neural networks, which deliver analytic or sampled predictive variances^23^. Once such a predictive distribution is available, conventional acquisition functions (maximizing predictive variance, expected improvement, or information gain) can be applied verbatim, so the additional challenge for regression lies not in the active-learning loop itself but in equipping the model with a trustworthy uncertainty estimator.

EMCM is a common AL strategy widely used in classification, regression, and ranking tasks. For regression tasks, several studies have developed AL algorithms that are based on EMCM ^25,26^. The goal of EMCM is to select the most informative unlabeled samples that induce the greatest model change, thereby improving the model’s learning efficiency and predictive performance.

Diversity in AL can refer to either the input space or the output space. Input space (IS) diversity aims to select samples that are well distributed in the feature space, encouraging the model to learn across the full data manifold ^27^, often implemented with distance-based or clustering methods. Output space (OS) diversity focuses on selecting samples whose predicted or actual target values differ from those already labeled, thus broadening the range of outcomes the model can capture. Considering both aspects helps AL select more informative and representative samples, improving model generalization.

Representativeness can be evaluated by the number of samples that are similar to or close to the target sample: the larger the number, the stronger the representativeness of the target sample. Obviously, the target sample should not be an outlier. In practical implementations, clustering methods are often used to identify the most representative examples ^28,29^. In some studies, representativeness is also estimated based on density; selecting an example from a high-density region of the domain space can increase the reliability of classification in its neighborhood ^30,31^.

**Table 1:** The table lists all AL strategies evaluated in this study, grouped into four categories: baseline, model-free, ML based, and DL based. Each strategy is described by its category and core principle. IS refers to input space, OS to output space, and EMCM to Expected Model Change Maximization. Some strategies incorporate multiple sampling criteria, including diversity, representativity, and uncertainty estimation.

Strategy	Strategy category	Principle
Random Sampling	baseline	Randomly
GSx	model-free	IS Diversity (Greedy, Distance-based)
EGAL	model-free	IS Diversity & Representativity
GSBAG	ML based	EMCM
GP	ML based	uncertainty estimate (Bayesian inference)
QueryByCommittee (QBC)	ML based	uncertainty estimate (Vote-entropy)
Tree-Based-D	ML based	IS Diversity (Tree partitioning)
Tree-Based-R	ML based	Representativity (Tree clustering)
QDD	ML based	Representativity & uncertainty estimate & IS Diversity
GSy	ML based	OS Diversity
iGS	ML based	IS Diversity & OS Diversity
RD-B	ML based	IS Diversity & representativity
RD-QBC	ML based	IS Diversity & uncertainty estimate
RD-GS	ML based	IS Diversity (Two-stage diversity via clustering and distance)
RD-EMCM	ML based	IS Diversity & EMCM
MCDO	DL based	uncertainty estimate (Approximate Bayesian Inference)
LL4AL	DL based	Loss Prediction
LCMD	DL based	IS Diversity & Representativity

All strategies, as well as their types and the principles they rely on, are shown in Table 1. Below is a concise summary of each tested AL strategy tested in this study, along with key sources and parameter settings, additional details for each strategy are presented in the supplementary Appendix A:

- **Random sampling**: Baseline strategy for selecting samples randomly.
- **EGAL**^30^: Model-free, purely exploratory method selecting samples based on density and diversity using similarity measures without model retraining. Details in Appendix A0.1.
- **GS-BAG**^32^: Greedy sampling method specifically for Gaussian Process Regression, selecting samples that maximally reduce model uncertainty. The kernel configuration combines RBF, Constant, and WhiteKernel with 10 random restarts for hyperparameter optimization. Details in Appendix A0.2.
- **GP**^23,33^: Utilizes predictive variance from Gaussian Process Regression to select samples with maximum uncertainty, employing the same kernel settings as GS-BAG. Details in Appendix A0.3.
- **Query by Committee (QBC)**^34^: Variance among predictions from a diverse 100-member committee (XGBoost, MLP, k-NN, Bayesian Ridge) is used to identify uncertain samples. Details in Appendix A0.4.
- **RT-AL**^35^: Regression tree-based strategies leveraging diversity and representativity principles, using distance and clustering to ensure comprehensive sampling. Details in Appendix A0.5.
- **QDD**^36^: Combines Query-by-Committee with diversity and density constraints, using equal weights for uncertainty, diversity, and density measures. Details in Appendix A0.6.
- **GSx/GSy/iGS**^37^: Greedy sampling approaches focusing separately on input space diversity (GSx), output space diversity (GSy), and jointly on both spaces (iGS). Details in Appendix A0.7.
- **RD-ALR**^38^: Integrates representativeness and diversity via clustering (RD), and further integrates QBC, EMCM, or Greedy Sampling (RD-QBC, RD-EMCM, RD-GS). Details in Appendix A0.8.
- **MCDO**^39^: Estimates uncertainty using Monte Carlo Dropout with an MLP architecture having two hidden layers (50 and 25 neurons), batch size 32, learning rate 0.01. Details in Appendix A0.9.
- **LL4AL**^40^: Predicts loss for unlabeled samples using a neural network, guiding selection towards samples likely to improve model performance, maintaining the neural network settings consistent with MCDropout. Details in Appendix A0.10.
- **LCMD**^41^: Combines clustering and maximum-distance sampling within the largest data cluster, using the same neural network parameters as MCDropout. Details in Appendix A0.11.

## Experiments

To comprehensively evaluate the effectiveness, reliability, and generalization capability of various active learning (AL) strategies in small-sample regression for materials science, we derived 13 single-task regression problems from 9 datasets involving many real-world materials, and conducted systematic experiments accordingly. This section describes the datasets used, the experimental setup in detail, and explains the evaluation metrics used for model performance assessment. To ensure fair and reproducible comparisons across strategies, consistent protocols are followed for model training, sampling and performance evaluation. Confidence intervals are also calculated to quantify variability and robustness under repeated trials.

### Datasets

The datasets selected for this study cover three representative material categories: concrete, metal, and composite. Each dataset represents one or more regression tasks involving structured, tabular data derived from expensive and time-consuming experiments. Such costly experimental settings are exactly where AL strategies can offer substantial value, as they aim to reduce experimental costs by selectively labeling only the most informative samples. Although a larger unlabeled data pool is ideal for thoroughly assessing AL performance, real-world materials datasets often suffer from limited sample sizes due to practical constraints. Consequently, these datasets provide realistic and challenging scenarios for evaluating AL methods in materials design, providing multidimensional support for materials performance prediction and intelligent mix design. Table 2 shows the overview for all datasets, where $R_{sf}$ is the ratio of the dataset size to the number of features.

**Table 2:** Datasets overview.

**Dataset Name**	**Domain**	**Size**	**Features**	$R_{sf}$
LI-2023 ^42^	Concrete	1110	16	69.4
UCI-concrete ^43^	Concrete	1030	8	128.8
Yin-2021 ^44^	Fibre reinforced polymers	922	11	83.8
Hu-2021 ^45^	Aluminum	896, 860, 783	27	33.2,31.9,29
RCFST ^46^	Rectangular Concrete-filled steel tube	622	7	88.9
UHPC_cs ^47,48^	Ultra-high-performance concrete	379	16	23.7
Matbench_steel ^49^	Steel	312	14	22.3
BFRC ^50^	Fiber-Reinforced Concrete	267, 245, 267	10	26.7,24.5,26.7
SHCC ^51,52^	Strain Hardening Cementitious Composites	240	37	6.5

**Fig. 2:**
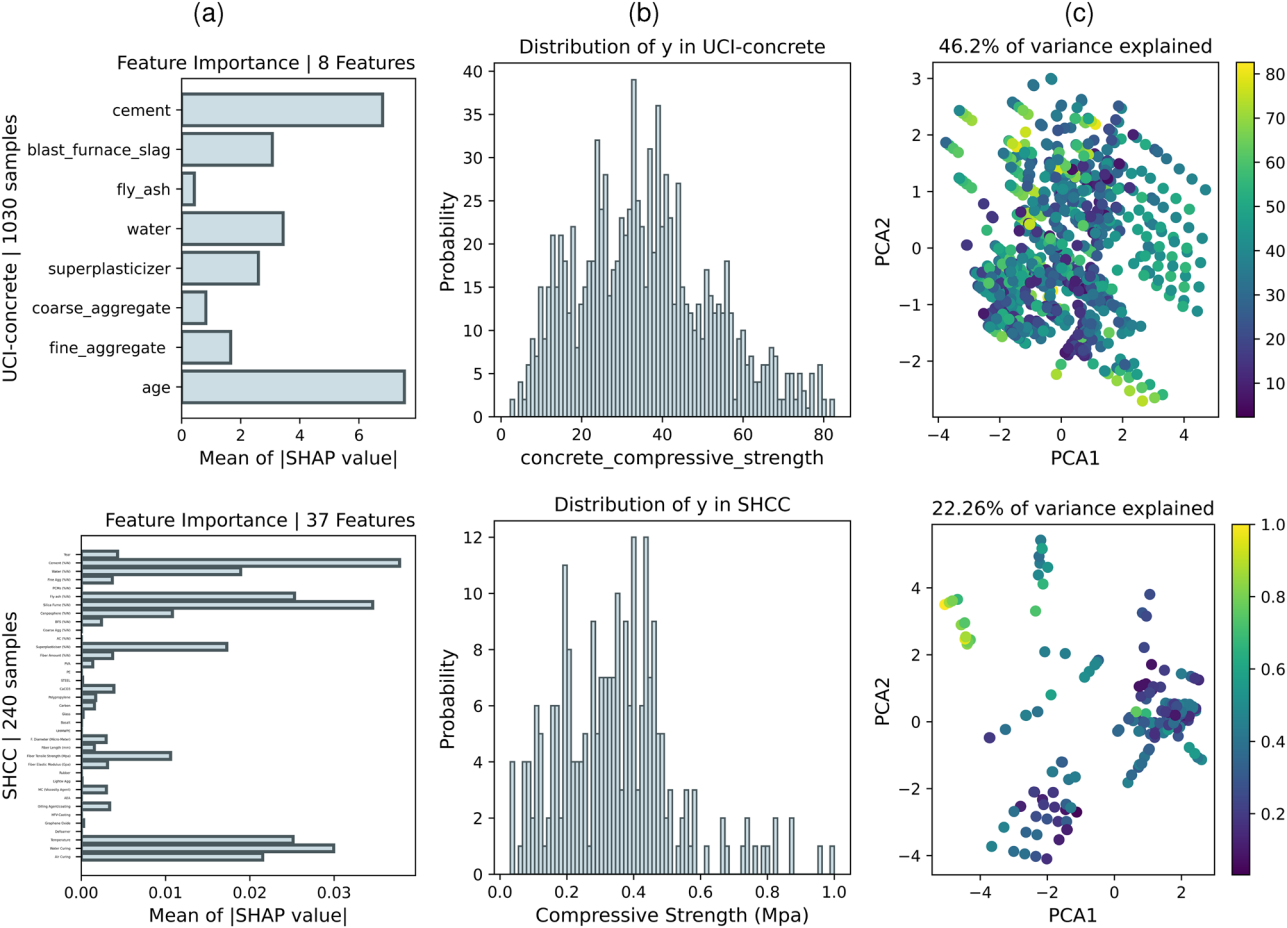
Design space visualisation from selected datasets. (**a**) Mean absolute SHAP values indicating the feature importance. (**b**) Histogram of the distribution of the target variable. (**c**) The visualization of the first two principal components represents the input feature space of the selected dataset, and the color of the dots represents the target values.

The LI-2023 dataset ^42^ comprises 1,110 concrete compressive strength data points collected from 39 studies, recording key parameters such as cement content, water-to-cement ratio, aggregate type, and gradation. The UCI-concrete dataset ^43^, a classical regression benchmark, includes 1,030 samples described by eight numerical features (e.g., cement, blast furnace slag, fly ash, water, superplasticizer, coarse aggregate, fine aggregate, and curing age) with a highly nonlinear relationship to compressive strength, serving as a platform for evaluating various regression models. The Yin-2021 dataset  ^44^ consolidates 922 fiber pullout test results (both experimental and simulation-based) with 11 variables characterizing fiber properties, matrix characteristics, and testing conditions to assess the interfacial mechanical properties of fiber-reinforced polymer composites. Yin-2021 was divided into Yin-2021-IFSS and Yin-2021-PF. The Hu-2021 dataset ^45^ contains 930 aluminum alloy samples sourced from handbooks and peer-reviewed literature, documenting key alloying element contents, processing parameters, and manufacturing characteristics (transformed via one-hot encoding) to support regression analysis. It was partitioned into Hu_UTS, Hu_YTS, and Hu_ELONGATION. The RCFST dataset ^46^ offers 622 experimental records of rectangular concrete-filled steel tube columns, featuring seven variables-including column dimensions, steel tube thickness, steel yield strength, concrete compressive strength, and loading eccentricity-that underpin structural capacity prediction. The UHPC dataset ^47,48^ comprises 1,228 ultra-high performance concrete samples covering 24 mix design variables, with a focus on 28-day compressive strength. The Matbench_steel dataset ^49^, part of the Matbench benchmark suite, provides steel compositions (expressed in atomic fractions) alongside corresponding yield strengths, facilitating the evaluation of ML models in predicting mechanical properties. The BFRC dataset ^50^, extracted from numerous studies, concentrates on 28-day test data for basalt fiber-reinforced concrete, including mix design variables and fiber characteristics including compressive strength, flexural strength, and splitting tensile strength. It was divided into BFRC_CS, BFRC_FS, and BFRC_STS. Finally, the SHCC dataset ^51^ has 38 parameters, including mix design variables (e.g., cement, supplementary materials, aggregates, fibers) and other experimental conditions-to comprehensively describe the multidimensional performance of strain-hardening cementitious composites. For the SHCC dataset, the first eight target variables are treated as features to predict the final target variable “Compressive Strength (MPa)”, thereby simulating an extreme scenario characterized by high dimensionality and a small sample size.

Fig. 2 presents visualizations of two representative datasets (UCI-concrete and SHCC), summarizing the distributional diversity observed across all datasets in this study. Fig. 2a shows the SHAP-based feature importance analysis: in UCI-concrete, a small number of features dominate model predictions, whereas SHCC displays a more dispersed and generally weaker feature importance profile, indicative of high-dimensional sparsity. Fig. 2b depicts the distribution of the target variable for each dataset. Even after standardization, substantial differences remain: UCI-concrete exhibits a relatively smooth target distribution, while SHCC demonstrates a highly imbalanced and skewed target distribution. Fig. 2c further reveals the intrinsic structure via principal component analysis (PCA), with sample points colored according to their target values. Additionally, the variance explained by the first two principal components is annotated in Fig. 2c for each case. Collectively, these visualizations reflect the pronounced heterogeneity of tabular materials datasets in terms of feature relevance, target balance, and intrinsic structure, highlighting the challenges faced by AL methods in generalizing across diverse data scenarios. Notably, PCA and SHAP provide qualitatively consistent signals about feature relevance: for UCI-concrete, the clear colour gradient of the target along the first two PCs (46.2% variance explained) indicates that the response varies mainly within a low-dimensional, high-variance subspace, which accords with the SHAP finding that a few variables (e.g., cement, water, age) dominate. In contrast, for SHCC the first two PCs explain only 22.26% of variance and no monotonic target trend is apparent in the PCA plane, consistent with the small and diffuse SHAP magnitudes, i.e., high-dimensional sparsity.

### Experimental setup

To ensure reproducibility and reliability of the results, all aspects of experimental design and implementation were rigorously controlled. First, in order to obtain statistically significant confidence intervals and maintain consistent reproducibility, each AL strategy was tested with 20 independent experiments, using random seeds fixed from 30 to 49. All other conditions remained the same across different experimental runs, thus guaranteeing a fair comparison of performance under identical settings.

To approximate the conditions of large-scale materials experiments, the sampling size per iteration was set to 10. Since no pre-trained model was available at the initial sampling stage, all model-based AL strategies defaulted to random sampling in the first iteration. From the second iteration onward, the strategies used the model trained in the previous iteration to select additional unlabeled data, continuing this process until the entire dataset was labeled.

From an implementation perspective, all DL-based methods (*e.g.*, MCDropout, LL4AL, and BMDAL) employed the same network architectures and hyperparameters shown in Table 3. The network structure, model settings and boundary conditions are based on the MLP architecture designed by Li *et al.* ^53^ in concrete compressive strength prediction tasks to ensure robust nonlinear fitting capabilities and efficient training.

**Table 3:** Architecture and hyperparameters of the multi-layer perceptron (MLP) model applied to all DL-based methods.

Parameter name	Parameter value
Input Layer	input feature nodes corresponding to experimental variables
Hidden Layer 1	50 neurons with ReLU activation
Hidden Layer 2	25 neurons with ReLU activation
Output Layer	Single output neuron for regression with linear activation
Batch size	32
Learning rate	0.01

For Gaussian process (GP)-based strategies, such as GP and GABAG, the same kernel function and core components were employed to eliminate confounding effects resulting from differences in model structures. Based on insights from multiple studies that applied GPR models to material science datasets^54–56^, the following kernel combination was selected:

- **RBF (Radial Basis Function, Squared Exponential Kernel):** Captures smooth, nonlinear relationships in material data.
- **C (Constant Kernel):** Scales the overall output of the kernel function.
- **WhiteKernel (White Noise Kernel):** Models measurement noise or experimental errors in the data.

Additionally, to enhance the model’s global search capability and prevent it from getting trapped in local optima, multiple random restarts of the optimizer (n_restarts_optimizer = 10) were applied. This approach conducts multiple rounds of hyperparameter optimization from different initial values, further improving model performance and robustness.

The Auto-Sklearn ^57^ framework was selected as the AutoML approach for this study, owing to its superior performance on small-sample datasets typical of materials design, as reported in previous works^9^. To further ensure fairness, all Auto-Sklearn models trained by different AL strategies were allotted the same maximum fitting time of 300 seconds, and with $R^2$ consistently used as the evaluation metric for guiding model selection and hyperparameter optimization. Additionally, the model was validated using a five-fold cross-validation scheme, with all other training hyperparameters kept at their default values.

To assess the potential impact of a uniform time budget on models of different complexity, we first audited the max single-fit time enforced by Auto-Sklearn for all base learners/pipeline variants observed in our runs: from about 1.5 s for DecisionTreeup to about 29.0 s for ARDRegression, with none exceeding approximately 29 s. Consequently, under overall budgets of 150/300/600 s per fit, each search can evaluate a diverse set of configurations, including relatively high-complexity models; a uniform budget therefore does not systematically disadvantage complex pipelines. Furthermore, to verify that our conclusions do not hinge on a particular budget, we conducted a budget-sensitivity sub-study on three representative datasets (Hu-2021_ELONGATION, Matbench_steel, UCI-concrete) and two strategies (iGS, RD-EMCM): after each query we refit Auto-Sklearn with 150 s, 300 s, and 600 s budgets and recorded test-set $R^2$ learning curves. As shown in supplementary Appendix E, Fig. 12, the trajectories under the three budgets are nearly indistinguishable across all datasets and both strategies, with only minor fluctuations in the earliest iterations that do not alter the relative ordering of strategies. Taken together, these results indicate that using a fixed 300 s fitting budget ensures resource parity without introducing systematic bias into our conclusions regarding the comparative effectiveness of the sampling strategies.

With respect to hardware, all experiments were conducted on the same type of virtual machine in an exclusive manner. Each virtual machine was equipped with 10 independent CPU cores (Intel Xeon Platinum 8470 at 2.00 GHz) and 20 GB of available memory. Memory usage was not observed to be a limiting factor, thereby eliminating bias due to potential hardware resource constraints.

With respect to data management, all datasets were stored as CSV files and processed automatically based on a unified metadata file. For multi-output tasks, each output was isolated into a single-output problem to minimize potential interactions across multiple labels. This multifaceted and careful design ensured that the proposed AL strategies were evaluated under small sample material design conditions that emphasized reproducibility, fairness, and reliability, thus providing a strong foundation for subsequent analysis of the results.

### Evaluation metrics

Two metrics were used in this study to evaluate the regression model produced by AutoML: **Mean Absolute Error **(*MAE*) and **Coefficient of determination **($R^2$). *MAE* gives the average absolute difference between the predicted and true values, offering a direct measure of the prediction error in the same units as the target variable. The $R^2$ score reflects the proportion of the variance in the observed data that is explained by the model. A value closer to 1 indicates a better fit. These metrics provide complementary insights into model performance by quantifying prediction errors from different perspectives and are calculated as:

1 $$\begin{aligned} \text {MAE}= & \frac{1}{n} \sum _{i=1}^{n} \left| y_i - \hat{y}_i \right| \end{aligned}$$

2 $$\begin{aligned} R^2= & 1 - \frac{\sum _{i=1}^{n} (y_i - \hat{y}_i)^2}{\sum _{i=1}^{n} (y_i - \bar{y})^2} \end{aligned}$$

Here, $y_i$ represents the actual value; $\hat{y}_i$ the predicted value; $\bar{y}$ the mean of the observed values; and $n$ the total number of samples. Together, these metrics allow for a comprehensive assessment of model accuracy and reliability.

This study also evaluates the performance of AL strategies. After each round of sampling and training, the performance of the model on the test set was recorded, resulting in a performance curve related to the number of iterations. For example, the efficiency of different AL strategies in reducing *MAE* can be quantified by calculating the area between the performance curve and the horizontal axis, which is called Area Under the Curve (*AUC*). For the same number of iterations, if the *MAE* can be reduced faster, the area under the curve (AUC) will be smaller, indicating higher efficiency with a fixed labeling budget.The *AUC* is calculated as follows:

3 $$\begin{aligned} \textrm{AUC} = \int _{t_{\min }}^{t_{\max }} \textrm{MAE}(t)\,\textrm{d}t, \end{aligned}$$

where $t$ denotes the index of iteration or labeling cost, $\textrm{MAE}(t)$ is the mean squared error of the model at iteration $t$, and $t_{\min }$ and $t_{\max }$ are the start and end points (in terms of iteration or labeling cost) over which the *MAE* is measured.

### Definition of confidence interval

Based on the work of Colas *et al.* ^58^, at least 20 repeated experiments are required to reliably estimate the variability of algorithm performance. Therefore, as described in the Experimental Setup section, 20 tests with different random seeds are conducted for each strategy. The single-sample *t*-test is then employed to calculate the margin of error $\Delta$ and the confidence interval **CI** for these experimental results:

4 $$\begin{aligned} {\Delta }= & t_{\alpha /2,\,n-1} \times \frac{s}{\sqrt{n}} \end{aligned}$$

5 $$\begin{aligned} {\textbf {CI}}= & \bar{x} \;\pm \; \Delta \end{aligned}$$

where $\bar{x}$ denotes the mean value of the performance metric, *s* is the standard deviation of the performance metric, and *n* is the number of samples (in this context, $n=20$). After specifying the significance level $\alpha$, the two-tailed critical value $t_{\alpha /2,\,n-1}$ is obtained from the *t*-distribution table or determined using a statistical function, given the degrees of freedom $n - 1$. Based on Equation Eq. (5), the confidence interval can then be estimated from 20 independent experiments, providing a more reliable reflection of the variability in the algorithm’s performance metric under different random seeds and offering a more objective basis for comparing different algorithms or strategies.

## Results

This section systematically compares and analyzes the performance of various AL strategies across multiple datasets. Specifically, 18 strategies introduced in the Methods section-each based on distinct theoretical principles-are applied to 14 single-output regression tasks derived from the 9 materials datasets described in the Datasets section. The performance of these strategies is first examined over the course of labeling the entire unlabeled data pool, tracking how model performance evolves as additional data points are labeled. This approach reveals the strategies’ convergence speed and generalization capabilities. Subsequently, considering real-world constraints such as cost and practicality in materials design, the analysis focuses on strategy performance within the range of 60% to 90% of the maximum $R^2$ score. Early comparisons in this range are based on the fact that obtaining very high model accuracy does not necessarily yield commensurate benefits in most development processes. Moderate levels of prediction accuracy are often sufficient for the majority of materials design needs. Therefore, this comparison of strategies within this performance range not only highlights their effectiveness under limited labeling budgets but also offers a more realistic assessment for industrial applications, providing valuable insight for selecting suitable AL strategies.

### Overall performance comparison

**Fig. 3:**
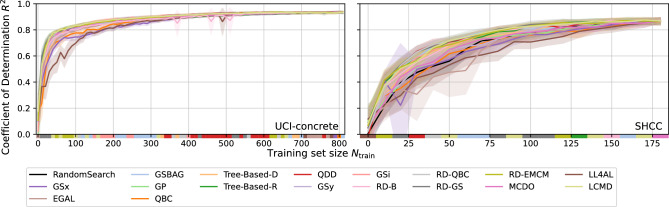
$R^2$ score trends for all tested AL strategies on selected datasets. The color bar shows the colors represented by the AL strategies that had the best model performance (maximum $R^2$ score) for a given training set sample size.

Fig. 3 shows the $R^2$ scores in two single-task scenarios (UCI-concrete and SHCC), while the remaining results are provided in Fig. 10 and Fig. 11 of supplementary Appendix D. In each subplot, the vertical axis (y-axis) represents the model’s $R^2$ score on the test set, and the horizontal axis (x-axis) shows the size of the labeled dataset. Each strategy is shown with a line and a shaded region: the line indicates its average performance from multiple runs, and the shaded region represents the range of possible variation, which reflects the strategy’s stability and any uncertainties. Overall, the strategies follow similar patterns. However, when the labeled dataset size is the same, the $R^2$ scores can still be different. This suggests that each strategy has its own strengths in choosing data and training models, and that AL can make decisions suited to specific tasks. To compare their performance more clearly, a color bar is placed below each plot. This bar shows which strategy achieves the highest $R^2$ score at different dataset sizes, making it easier to see the relative advantage of each strategy.

For the Matbench_steel dataset, when the number of labeled samples is at most 40, the AutoML model that is trained with the RD-B strategy consistently achieves the highest $\mathrm {R^2}$ score. When the number of samples is between 40 and 110, the Tree-based-R strategy demonstrates optimal performance. From 140 to 180 samples, the GSBAG strategy provides the best outcome for the AutoML. A similar approach can be employed to compare performance on other datasets.

In this study, the large number of AL strategies considered renders it challenging to directly observe the overall trends of each strategy. To address this issue, *AUC* (Area Under the Curve) is introduced as an evaluation metric, as described in the Evaluation Metrics section. *AUC* represents the area under the curve and is used in this work to quantify the region enclosed between the performance curve of AL strategies and the X-axis. Furthermore, to enhance comparability among different strategies, a normalized representation is adopted. Specifically, for each dataset, the *AUC* value of each AL strategy is computed and compared with the *AUC* of the baseline strategy (Random Search) by calculating their *AUC* ratio. This normalization allows for a fair comparison of strategy performance across different datasets, providing a clearer understanding of the relative strengths and limitations of each strategy, given by:

6 $$\begin{aligned} \textrm{AUC}_{\textrm{rel}} = \frac{\textrm{AUC}_{\textrm{Strategy}}}{\textrm{AUC}_{\textrm{RandomSearch}}} \end{aligned}$$

**Fig. 4:**
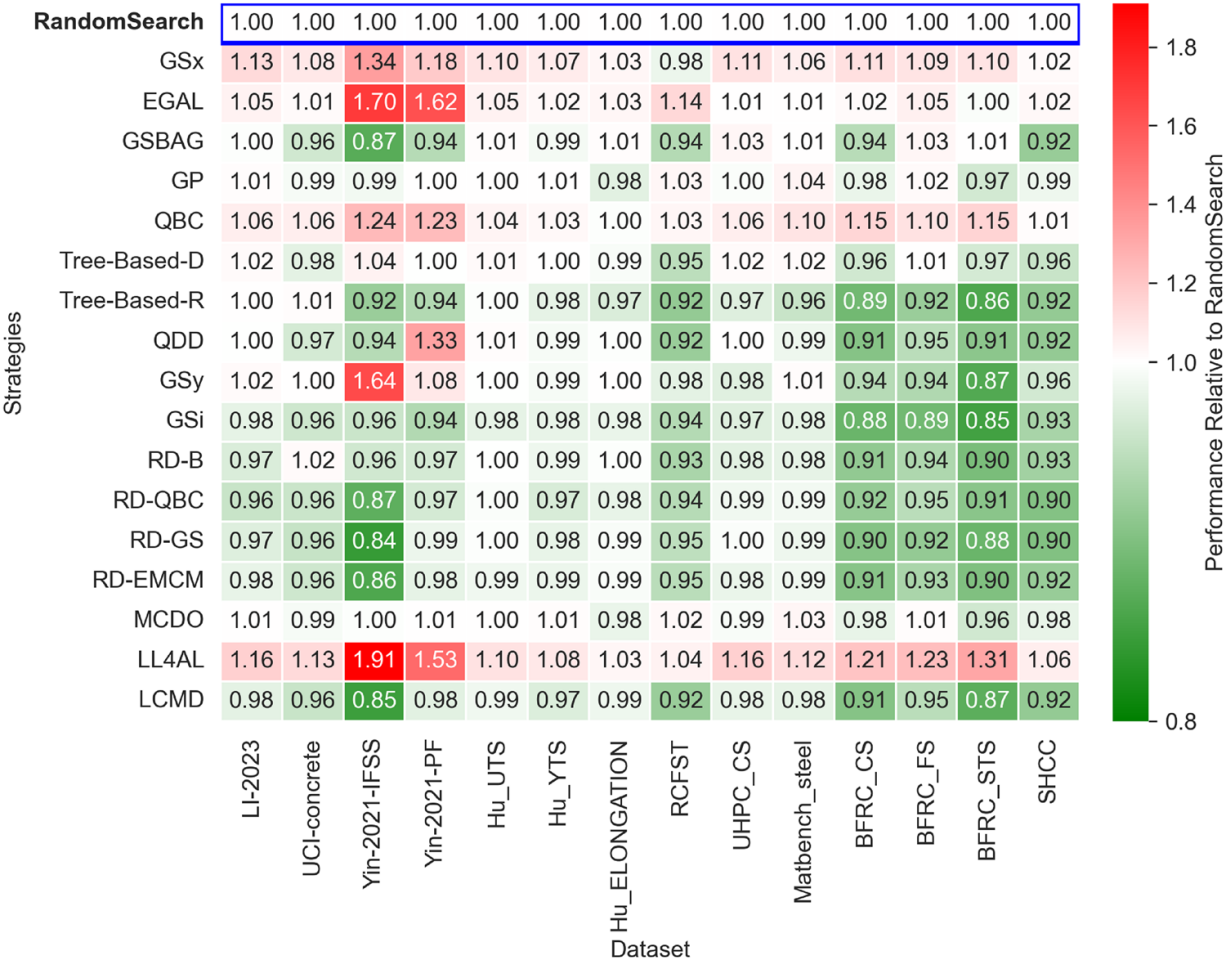
Relative *AUC* scores of the *MAE* learning curves reached by each AL strategy on each dataset. RandomSsearch was the baseline, set to 1.

Fig. 4 shows the *AUC* scores of the *MAE* performance curves for all AL strategies across all datasets. In the figure, red indicates *AUC* scores higher than those of the baseline strategy, while blue indicates scores lower than those of the baseline. Since *MAE* is highly robust, it is more suitable than other error measures as an evaluation metric for the complete performance curve. In AL tasks, the goal of an AL strategy is to reduce the *MAE* as much as possible-that is, to achieve as small an *AUC* score as possible-thereby demonstrating the strategy’s ability to lower the error more quickly. Therefore, for the *MAE* performance curve, a smaller *AUC* score indicates a better performing AL strategy.

Overall, the performance of the strategies shows that both GSx and EGAL perform worse than random search across all datasets. This suggests that model-free strategies, which rely solely on distance calculations, are not suitable for the AutoML framework in material design datasets. Specifically, GSx uses Euclidean distance to evaluate the diversity of the input space, while EGAL computes the cosine similarity of samples to measure representativeness and diversity. However, both methods depend on the feature distribution of the unlabeled data to differentiate samples and do not sufficiently consider the impact of samples on model learning. As noted in the Dataset section, different features contribute differently to and have varying sensitivities with respect to the target variable. The relationship between features and targets can be highly non-linear in some regions and with little influence in other regions. So just equal sampling from input space cannot adjust to such behavior. This observation reveals a limitation of model-free AL strategies in this study: selecting high-value samples requires not only consideration of the input feature space but also the sample’s actual effect on the model learning process. In other words, the effectiveness of an AL strategy largely depends on the soundness of its sample selection criteria and focusing solely on the geometric distribution of the input space is insufficient.

**Fig. 5:**
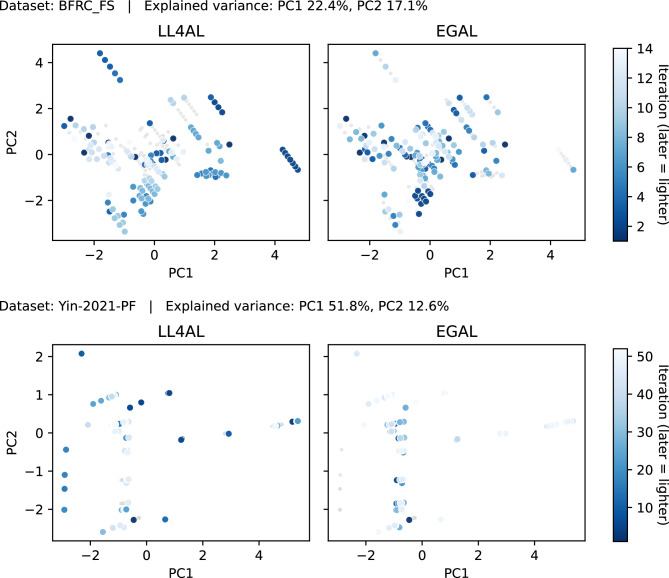
Acquisition trajectories on the PCA plane (first 70% of queries).

Furthermore, AL strategies based on deep neural networks (DNNs) did not perform well in this study, which aligns with recent findings by Grinsztajn *et al.* ^59^ that neural networks consistently underperform compared with tree-based models on small- to medium-sized tabular datasets because of unfavorable inductive biases and sensitivity to irrelevant features. Among these, LL4AL had the worst sampling performance across all datasets, and the MCDO strategy, which is based on simple uncertainty estimation, was slightly inferior to the baseline method. LL4AL was originally designed for classification tasks, with the core idea of building an auxiliary model to predict the loss of the main model on the unlabeled dataset, and then selecting the samples that would most affect the model. However, Yoo *et al.* ^40^ pointed out that using *MSE* to regress the loss function does not work well, possibly because a sample’s impact on the model is not solely determined by its loss. Some samples that are expected to cause high loss might do so because they provide valuable information, or because they contain high noise that misleads the model training. Thus, this loss-prediction based AL strategy has certain limitations in regression tasks. On the other hand, MCDO relies on the randomness of neural network weights to simulate Bayesian uncertainty estimation. However, this method can lead to unstable uncertainty estimates, which in turn affects the reliability of sample selection. Atighehchian *et al.* ^39^ noted that MCDO performs better on large datasets, but its performance on small datasets is not as prominent. Since the datasets in this study are relatively small, this may further exacerbate the instability of the MCDO strategy, thereby affecting its performance in AL scenarios.

To examine why some strategies (e.g., LL4AL) perform worse, we add acquisition-trajectory plots for LL4AL and EGAL on BFRC_FS and Yin-2021-PF (Fig. 5). We show only the first 70% of acquisitions, because most inter-strategy differences appear in the early half of the sampling budget. Each plot shows the PCA plane of the first two components (titles report explained variance); points are coloured from dark (early) to light (late). On BFRC_FS, LL4AL quickly concentrates in a few local clusters, and later iterations remain nearby, leaving large regions of the plane unexplored. EGAL covers the plane more evenly at first but often returns to peripheral areas, resulting in visible repetition. On Yin-2021-PF, the data form several bands along PC1. LL4AL remains within a narrow band for many iterations, whereas EGAL crosses several bands but again revisits outer areas. In small-sample settings with an AutoML surrogate that may change across iterations, LL4AL tends to prioritise hard but low-utility points within the same cluster; EGAL relies only on geometric spread and ignores output-related structure, which leads to redundant selections. These qualitative observations are consistent with the AUC results.

In contrast, the LCMD algorithm performed excellently on all datasets and was significantly better than random search. LCMD uses a gradient kernel to measure the similarity of samples in the neural network parameter gradient space and combines the principles of representativeness and diversity in sample selection. Compared with other DL-based AL strategies (such as LL4AL and MCDO), LCMD takes the neural network’s internal learning mechanism into account more fully by directly evaluating the influence of samples from the perspective of gradient information. This method effectively avoids the issues associated with relying solely on loss prediction or uncertainty estimation, and it demonstrates more stable and superior performance on the datasets in this study. These findings suggest that, within a DL framework, designing AL strategies that incorporate changes in internal model parameters (such as gradient information) may be more advantageous than traditional uncertainty estimation methods, especially when data is limited.

For AL strategies based on ML models, the overall performance met expectations. The Auto-Sklearn itself is built on ensemble learning algorithms that integrate multiple ML models, so it was assumed at the beginning of the experiments that all AL strategies based on ML models would show certain advantages. To further investigate the model-switching behavior of Auto-Sklearn during the active learning loop, we compared three datasets of varying complexity: UCI-concrete (low-dimensional, relatively simple), Matbench-steel (medium-dimensional, moderate complexity), and Hu-ELONGATION (high-dimensional, complex). These datasets were chosen to represent typical scenarios in small-sample materials science regression with different feature dimensionalities and structural characteristics. We tracked the dominant base learner selected by Auto-Sklearn ensembles after each iteration. The results (supplementary Appendix C, Fig. 9) show that Auto-Sklearn’s model preferences are strongly dependent on dataset complexity. On the UCI-concrete dataset, Auto-Sklearn rapidly converged to Gradient Boosting with minimal switching. On the Matbench-steel dataset, Auto-Sklearn initially explored multiple learners but quickly stabilized on Extra Trees as more samples became available. In contrast, on the Hu-ELONGATION dataset, Auto-Sklearn continued to alternate between different learners throughout the AL iterations. These findings demonstrate that AL strategies must remain effective under dynamically evolving model families, underscoring the importance of benchmarking AL methods in an Auto-Sklearn setting rather than assuming a fixed learner.

However, the performance of some strategies was anomalous. For example, the classic Query by Committee (QBC) strategy performed worse than random search on all datasets did, which contrasts with some previous studies. This may be because QBC relies solely on the inconsistency among the committee models to select samples, and it does not fully consider the representativeness and diversity of the samples. In contrast, the RD-QBC strategy performed excellently on all datasets. Its main difference is that, in addition to using the committee query, it also combines the principles of representativeness and diversity, thereby selecting samples with greater learning value more effectively. Moreover, in the RD variant strategies, RD-B is used as the base algorithm, and the other three RD variants integrate additional sampling principles (such as uncertainty measurement and the EMCM method). The experimental results show that these RD variants improve to varying degrees compared to the base algorithm. For example, on the UCI-concrete dataset, the *AUC* scores of the three RD variants were significantly lower than those of RD-B, indicating that in cases of complex data distributions, AL strategies that combine multiple principles can select high-value samples more effectively.

From the perspective of datasets, not all datasets can benefit from AL strategies to improve model training efficiency. Even the well-performing RD strategy and Tree-Based-R strategy did not significantly outperform the baseline random search on the Hu-2021, Li-2023, and Matbench_steel datasets. This phenomenon suggests that the characteristics of the dataset itself may affect the effectiveness of AL strategies, especially when the data distribution is complex, the relationship between features and the target variable is weak, or the dataset has a high level of noise. Specifically, when the dataset has high feature dimensions but few important features, AL strategies may find it difficult to distinguish valuable samples effectively. In addition, in some material design datasets, the dependency of the target variable on the input features may have a strong nonlinear structure, and some AL strategies may not be able to fully capture these complex relationships, leading to a decline in sampling quality.

### Staged performance comparison

**Fig. 6:**
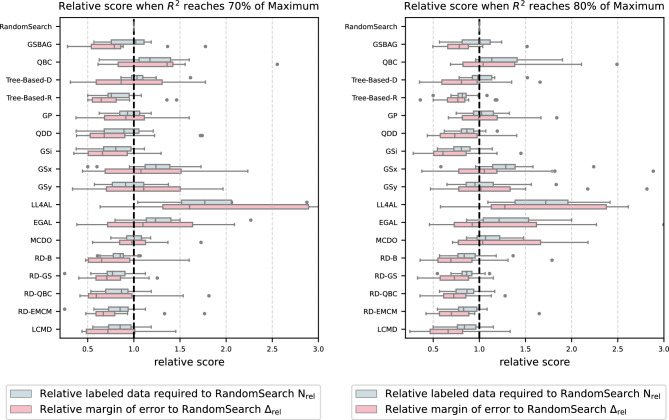
Distribution of the relative score ($\textrm{N}_{\textrm{rel}}$, $\mathrm {\Delta }_{\textrm{rel}}$) of each AL strategy when reaching 70% and 80% of the maximum $R^2$ score.

Therefore, in this study, When the $R^2$ score of the AutoML model reaches 60%, 70%, 80%, and 90% of its maximum value, the ratio of labeled data required by different active learning strategies-relative to the baseline (random search)-is recorded. This ratio is denoted by $N_{\textrm{rel}}$, and the ratio of the margin of error (confidence interval) is denoted by $\Delta _{\textrm{rel}}$. They are defined as follows:

7 $$\begin{aligned} \textrm{N}_{\textrm{rel}}= & \frac{\textrm{N}_{\textrm{Strategy}}}{\textrm{N}_{\textrm{RandomSearch}}} \end{aligned}$$

8 $$\begin{aligned} \mathrm {\Delta }_{\textrm{rel}}= & \frac{\mathrm {\Delta }_{\textrm{Strategy}}}{\mathrm {\Delta }_{\textrm{RandomSearch}}} \end{aligned}$$

Values of $N_{\textrm{rel}}$ and $\Delta _{\textrm{rel}}$ greater than 1 indicate that the strategy is worse than the baseline. Fig. 8 in supplementary Appendix B and Fig. 6 present the distribution of relative scores for each AL strategy. The distribution associated with each strategy is derived from its performance across all datasets, thereby providing a comprehensive representation of the strategy’s overall effectiveness under varying data characteristics.By examining the distribution of $N_{\textrm{rel}}$, one can evaluate the efficiency of each strategy in terms of labeled data usage. A lower median and a more concentrated distribution indicate that the strategy requires fewer labeled samples to reach the target performance, reflecting higher efficiency.The distribution of $\Delta _{\textrm{rel}}$, on the other hand, serves as an indicator of the stability of each strategy across different datasets and random seeds. A smaller margin of error suggests more consistent performance and stronger robustness, while greater variability implies that the strategy is more sensitive to randomness and variations in data distributions.

In general, as shown in Fig. 6, the comparison of the average data required and the margin of error when reaching 70% and 80% of the maximum $R^2$ score is largely consistent with the results of the *AUC* comparison. The results for 60% and 90% of the maximum $R^2$ score are shown in the supplementary Appendix B.

Specifically, when 70% of the best $R^2$ score is reached, model-free AL strategies based on diversity and representativeness (such as GSx and EGAL), AL strategies designed for DL (such as MCDO and LL4AL), and the model-based QBC strategy based on committee query performed the worst. The required amount of sampled data, $N_{\textrm{Strategy}}$, was generally greater than that of the baseline, and the margin of error was also noticeably larger. In contrast, the ML-based AL strategies, Tree-based-R (based on representativeness) and RD-GS (based on the diversity of the input space), not only had $N_{\textrm{rel}}$ and $\Delta _{\textrm{rel}}$ values less than 1 but also exhibited a more concentrated data distribution, showing particularly excellent performance.

When reaching 80% of the best $R^2$ score, the model-free strategies GSx and EGAL, along with the DL-based strategies MCDO and LL4AL, still performed poorly; however, Tree-based-R, the RD-EMCM strategy that integrates diversity with EMCM across domains, and the GSi strategy based on output space diversity demonstrated exceptionally good performance.

It is worth noting that when 70% of the best $R^2$ score is reached, the GSy strategy which is based on output space diversity required less data than the baseline, yet its relative $\mathrm {\Delta }_{\textrm{rel}}$ was greater than 1. This indicates that although the strategy achieved higher average performance on most test datasets, its predictive confidence was lower, and its performance was less stable.

### Verification of the surrogate MLP performance

Notably, in our benchmark experiments, several deep learning (DL)-based active learning strategies, particularly LL4AL, substantially underperformed compared to the baseline across a majority of the datasets. To ascertain whether this poor performance could be attributed to an inadequate predictive capability of the underlying MLP architecture, which serves as the surrogate model for these strategies, a targeted verification experiment was conducted. For this purpose, we selected three representative datasets (Yin-2024-IFSS, UCI-concrete, and Hu-2021-UTS) and evaluated the performance of three MLP architectures of varying complexity. In addition to the original network architecture used throughout this study (a two-hidden-layer network with 50 and 25 neurons, respectively), two additional variants were tested: a shallower, less complex network consisting of a single hidden layer with 60 neurons, and a deeper, more complex network featuring a three-hidden-layer architecture with 50, 25, and 15 neurons, respectively. As shown in Table 4, all three MLP architectures demonstrated strong predictive capabilities on their own, achieving high $R^2$ scores in a standard regression setting. This indicates that our chosen MLP is a competent and robust base model. Consequently, the underperformance of strategies such as LL4AL should be attributed to the sampling strategy itself, rather than to a lack of predictive power in the base model. This verification further justifies our methodological choice of employing a fixed MLP architecture throughout the benchmark to ensure a fair and direct comparison of the AL strategies.

**Table 4:** Verification of MLP architectures’ performance on selected datasets. The evaluation metric is the $R^2$ (Coefficient of determination).

Dataset	Shallow MLP	Original MLP	Deep MLP
	(hidden_layers=(60,))	(hidden_layers=(50, 25))	(hidden_layers=(50, 25, 15))
Yin-2024-IFSS	0.997	0.997	0.997
UCI-concrete	0.874	0.887	0.886
Hu-2021-UTS	0.896	0.883	0.904

### Computational cost of active learning strategies

Since both AutoML and AL require iterative retraining, we additionally measured the *maximum computing time per single acquisition step* of each strategy across three representative datasets (Hu_ELONGATION, Matbench_steel, and UCI-concrete). While AutoML retraining (300 s per iteration) dominates the runtime, the acquisition functions themselves differ substantially in computational overhead.

- **Negligible cost **(<1 s): GaussianProcessBased, TreeBased-Diversity, EGAL.
- **Low cost **(1–5 s): GSBAG, TreeBased-Representativity, Basic-RD, RD-GS, mcdropout.
- **Moderate cost **(5–30 s): BMDAL, LearningLoss, GSx, GSy, QueryByCommittee.
- **High cost **(>60 s): QDD, iGS, RD-QBC.

Table 7 in supplementary Appendix F summarizes the maximum acquisition time observed for each method. Overall, model-free and simple tree/GP-based strategies add negligible cost, whereas committee-based and certain diversity-hybrid strategies (notably RD-QBC) are substantially more expensive. Importantly, since AutoML retraining dominates the total runtime, the relative differences among AL strategies are primarily relevant when many iterations are performed or when operating under strict compute budgets.

### Dataset specific results

To directly address dataset-level differences, Table 5 summarizes the relative data reduction achieved by the best-performing AL strategy compared to random sampling at 60%, 70%, 80%, and 90% of the maximum attainable $R^2$ on each dataset. To clearly illustrate the performance of the optimal strategy under specific application scenarios, we report only the strategy that achieved the greatest improvement for each dataset and target accuracy level. Substantial gains (above 30%) were observed for Yin-2021, BFRC, and SHCC, whereas datasets such as Hu_YTS and UHPC_CS exhibited only marginal improvements (below 20%). This finding highlights that AL is particularly beneficial when datasets exhibit strong non-linear structure and redundancy, but may yield limited advantage when feature–target relationships are weak or noisy.

**Table 5:** Relative data reduction achieved by the best-performing AL strategy compared to random sampling at different fractions (60%, 70%, 80%, 90%) of the maximum attainable $R^2$ on each dataset.

Dataset	60%		70%		80%		90%	
LI-2023	Tree-Based-R	26.4%	RD-QBC	36.3%	RD-QBC	31.8%	RD-QBC	39.0%
UCI-concrete	RD-GS	20.0%	RD-GS	22.7%	RD-GS	9.9%	LCMD	20.9%
Yin-2021-IFSS	RD-GS	85.7%	RD-EMCM	75.0%	GSx	41.7%	GSx	52.2%
Yin-2021-PF	RD-GS	58.8%	QDD	50.0%	LCMD	50.0%	RD-B	63.9%
Hu_UTS	GSi	37.9%	GP	16.7%	GP	9.7%	GSi	30.5%
Hu_YTS	RD-QBC	2.8%	RD-B	11.3%	RD-B	24.2%	LCMD	24.7%
Hu_ELONGATION	Tree-Based-R	22.2%	Tree-Based-R	24.7%	Tree-Based-R	17.8%	Tree-Based-R	22.1%
RCFST	GSi	18.2%	RD-B	12.5%	LCMD	20.0%	QDD	45.1%
UHPC_CS	GSy	9.6%	GSy	17.1%	Tree-Based-R	19.1%	RD-QBC	20.9%
Matbench_steel	Tree-Based-R	16.7%	Tree-Based-R	27.4%	RD-QBC	5.4%	Tree-Based-R	28.9%
BFRC_CS	QDD	41.8%	QDD	45.5%	GSi	39.4%	GSi	38.2%
BFRC_FS	GSi	37.0%	GSi	39.7%	GSi	26.0%	GSi	30.6%
BFRC_STS	GSy	42.4%	GSy	42.7%	GSy	35.0%	GSi	29.6%
SHCC	RD-GS	45.1%	RD-QBC	37.0%	QDD	37.9%	GSBAG	18.4%

## Conclusion

A comprehensive benchmark of diverse AL strategies for regression tasks in materials science was performed in this study, leveraging a unified framework across multiple heterogeneous datasets. These experiments compared methods that are based on uncertainty estimation, EMCM, and diversity and representativeness. They further show that strategies integrating multiple sampling criteria (for example, LCMD, the RD variants, and Tree-based-R) consistently outperformed the baseline random sampling approach in data-scarce scenarios. In contrast, model-free strategies and those solely based on geometric distances or DL uncertainty estimates tend to underperform, as they fail to capture the intrinsic structure of the data and the nuanced impact of individual samples on the model’s learning process. This observation underscores the critical importance of combining internal model feedback with external data characteristics to effectively guide the sampling process.Overall, this work provides a reproducible and extensible benchmark for regression-based AL in materials science, offering practical insights into the trade-offs between labeling cost and predictive performance. The study demonstrates that AL can significantly enhance model performance when data acquisition is expensive, thereby accelerating the materials design process.

Future research should explore the integration of AutoML with multi-strategy fusion to better address high-dimensional, complex data scenarios and further refine sample selection criteria. By leveraging these advancements, the field can move toward more efficient and cost-effective experimental designs, ultimately driving innovations in data-driven materials discovery and optimization.

## Supplementary Information

Supplementary Information.

## Data Availability

The datasets for the presented study are open access, see table 2 in paper for information on the individual datasets. The experimental setup and results are available on GitHub: https://github.com/bjhtud/Benchmark-AL-Mat.

## Code availability

The experimental setup is available on GitHub: https://github.com/bjhtud/Benchmark-AL-Mat.
