# Supplementary material for: A comprehensive benchmark of active learning strategies with AutoML for small-sample regression in materials science
Source: Sci Rep. 2025 Oct 23;15:37167. doi: 10.1038/s41598-025-24613-4 (PMC12550062; doi:10.1038/s41598-025-24613-4)
Supplement: Supplementary file 1 — Supplementary Information. [file 41598_2025_24613_MOESM1_ESM.pdf]

Appendix

A Comprehensive Benchmark of Active Learning Strategies with AutoML for Small-Sample Regression in Materials Science

Jinghou Bi, Yuanhao Xu, Felix Conrad, Hajo Wiemer, Steffen Ihlenfeldt
Dresden University of Technology DE, Faculty of Mechanical Science and Engineering, Dresden, 01069, Germany
Correspondence: jinghou.bi@tu-dresden.de

Overview

This supplementary document provides extended methodological details, additional figures, and computational analyses referenced in the main text. It contains Appendices A–F.

Appendix A: tested AL approaches

A0.1 EGAL

EGAL is a model-free AL strategy proposed by Rong Hu *et al.* [1]. It is a purely exploratory method that selects samples for labeling based solely on similarity measures, eliminating the need for classifier retraining. In this work algorithms are redesigned to be suitable for regression tasks.

EGAL primarily utilizes two metrics to guide sample selection:

1. Density

Density measures the compactness of a sample in feature space. It is computed as the sum of similarities of all neighbors within a predefined similarity threshold α:

$$\text{density}(x_i) = \sum_{x_r \in N_i} \text{sim}(x_i, x_r) \tag{9}$$

where N_i represents the neighborhood of x_i based on the similarity threshold α.

2. Diversity

Diversity measures how different a sample is from the currently labeled dataset. It is computed as the inverse of the maximum similarity with any labeled sample:

$$\text{diversity}(x_i) = \frac{1}{\max_{x_r \in L} \text{sim}(x_i, x_r)} \tag{10}$$

where L represents the set of currently labeled samples.

3. Candidate Selection

A similarity threshold β is used to ensure that selected samples are sufficiently different from labeled samples:

$$CS = x_i \in U \mid \text{sim}(x_i, x_j) \leq \beta, x_j \in L \tag{11}$$

where U is the pool of unlabeled samples.

EGAL computes density and diversity scores for all unlabeled samples and apply the similarity threshold β to filter candidates. then rank candidates based on density and select the highest-ranked sample for labeling. Finally, dynamically adjust β to maintain an appropriate exploration range.Repeat until the labeling budget is reached or a termination criterion is met.

A0.2 GS-BAG

GS-BAG (Greedy Sampling-based Batch AL for Gaussian Process Regression) is a batch AL method specifically designed for Gaussian Process Regression (GPR) by Zhao et al [2]. Its main idea is to measure the reduction in model uncertainty induced by new samples—using a concept akin to weight information gain—and to select those samples that contribute most to improving the model. Unlike the exhaustive search used in traditional BAG algorithms, GS-BAG employs a greedy strategy to select samples one by one, significantly reducing the computational cost while maintaining near-optimal performance.

In GS-BAG, for each candidate sample x, a selection criterion is defined as follows:

$$\pi^*(x; \theta, \sigma_\varepsilon^2, U^*, X_n) = k(x,x) + k(x, X_n \cup U^*)^T \left(K(X_n \cup U^*) + \sigma_\varepsilon^2 I\right)^{-1} k(x, X_n \cup U^*), \tag{12}$$

where:

- $k(x,x)$ is the self-covariance at x given by the kernel function,
- $k(x, X_n \cup U^*)$ denotes the covariance vector between x and the current training set (with inputs X_n) combined with the already selected samples U^*,
- $K(X_n \cup U^*)$ is the covariance matrix corresponding to the inputs $X_n \cup U^*$,
- θ represents the kernel parameters, and
- σ_ε^2 is the noise variance.

In the work by Zhao *et al.*[2], the Gaussian process (GP) kernels employed for the GS-BAG algorithm include the Exponential Kernel (EF), Matérn Kernel (M52), and Rational Quadratic Kernel (RQ). The performance of the GS-BAG algorithm primarily depends on the GPR model itself rather than the AL strategy, as evidenced by the similar results obtained with different kernels. Among these kernels, the Rational Quadratic Kernel (RQ) was predominantly used in subsequent experiments due to its stable performance across various datasets and application scenarios.

A0.3 GP

Gaussian Process Regression (GPR) is a widely used Bayesian non-parametric regression method in AL [3, 4]. In AL, the predictive variance of GPR is used to measure model uncertainty and guide the sampling strategy. For an unlabeled sample x^*, the predictive variance is given by:

$$\sigma_*^2 = k(x^*, x^*) - k_*^T (K + \sigma_n^2 I)^{-1} k_* \tag{13}$$

Based on this, the sample with the highest predictive variance is selected for AL to minimize model uncertainty and improve learning efficiency:

$$x_{\text{query}} = \arg\max_{x \in \mathscr{U}} \sigma_*^2(x) \tag{14}$$

In this study, this AL algorithm is reproduced based on this principle. In order to fix the boundary conditions, the kernel of the Gaussian process regression model used to measure the prediction uncertainty is set up as in the same way as used in Section **GS-BAG**.

A0.4 Query By Committee (QBC)

This work reproduces the Query by Committee (QBC) AL strategy described by Burbidge *et al.* [5]. A committee of 100 learners is implemented in a custom 'QueryByCommittee' class, with each learner initialized using randomized hyperparameters to promote diversity. Multiple regression models are employed, including **XGBoost**, **MLP**, **k-Nearest Neighbors**, and **Bayesian Ridge**, with hyperparameters sampled from grids using 'ParameterGrid'.

Following Burbidge *et al.* [5], uncertainty is measured using the prediction standard deviation from all committee members. For a sample x, let $\hat{y}_i(x)$ be the prediction from the i-th learner. The ambiguity $a(x)$ is defined as:

$$a(x) = \sqrt{\frac{1}{M} \sum_{i=1}^{M} (\hat{y}_i(x) - \bar{y}(x))^2} \tag{15}$$

where M is the number of learners (100), and $\bar{y}(x)$ is the committee average prediction:

$$\bar{y}(x) = \frac{1}{M} \sum_{i=1}^{M} \hat{y}_i(x) \tag{16}$$

The top n_{act} samples with the highest ambiguity are selected:

$$\text{NextSample} = \text{argsort}(a(x))[-n_{act} :] \tag{17}$$

This reproduction closely follows Burbidge *et al.* (2007) by using committee-based variance to identify informative samples. The implementation employs a custom committee of 100 diverse learners, enhancing variance-based sample selection performance.

A0.5 RT-AL

Jose *et al.* [6] proposed two regression tree-based AL algorithms designed to leverage both response and feature space information, enhancing sampling efficiency and model performance. Their method demonstrated strong performance, stability, and generalization across multiple benchmark datasets:

1. Diversity-based Query in Leaves: This method selects unlabeled samples farthest from the labeled ones within each regression tree leaf, improving data diversity and model generalization.

- **Distance Calculation**: Compute the Euclidean distance between each unlabeled sample and all labeled samples:

$$d_{ji} = \|x_j - x_i\|_2 \tag{18}$$

- **Sample Selection**: Select the unlabeled sample farthest from the labeled set:

$$j^* = \arg\max_j \min_i d_{ji} \tag{19}$$

- **Iteration**: Repeat until the desired number of samples is reached.

2. Representativity-based Query in Leaves: This method clusters unlabeled samples within each leaf and selects representative samples from each cluster to improve coverage and representativeness.

- **In-Leaf Clustering**: Use k-means clustering on the unlabeled samples.
- **Initial Selection**: Pick the sample closest to each cluster centroid.
- **Representativity Optimization**: Select samples balancing representativity and diversity:

$$j_\ell^* = \arg\max_j [\Delta(x_j) - R(x_j)] \tag{20}$$

Where:

$$R(x_j) = \frac{1}{|c_\ell| - 1} \sum_{x_m \in c_\ell, m \neq j} \|x_j - x_m\| \tag{21}$$

$$\Delta(x_j) = \min_{m \in I_{\text{init}} \cup \{j_k^*\}} \|x_j - x_m\| \tag{22}$$

A0.6 QDD

Kee *et al.* [7] developed an improved batch active learning (BAL) strategy, namely **QDD (Query-by-Committee + Diversity + Density)**. This method extends the **Query-by-Committee (QBC)** sampling approach by incorporating **diversity** and **density** to optimize instance selection. this method first employs QBC to compute sample uncertainty, using **predictive variance** as the uncertainty measure. Then, a **diversity constraint** is introduced to ensure the selected instances are well-distributed in the feature space, reducing redundancy. Finally, **density information** is incorporated to prioritize instances in high-density regions, improving representativeness and mitigating the impact of outliers. In this work, the number of committee members (i.e., learners) is set to **100**, as in the Query-by-Committee strategy. Furthermore, based on the findings in the original study, the weights for $f(x)$, $d(x)$, and $h(x)$ are set to **equal values**, i.e., $\lambda = \frac{1}{3}$, $\beta = \frac{1}{3}$.

The instance selection score function in QDD is defined as:

$$u(x) = (1 - \lambda - \beta) f(x) + \lambda d(x) + \beta h(x) \tag{23}$$

where: - $f(x)$ represents the uncertainty measure, computed using predictive variance:

$$f_{\text{var}}(x) = \frac{1}{|C|} \sum_{\theta \in C} (h_\theta(x) - \bar{h}(x))^2 \tag{24}$$

where C denotes the set of committee members, $h_\theta(x)$ is the prediction of model θ for instance x, and $\bar{h}(x)$ is the mean prediction across all committee members:

$$\bar{h}(x) = \frac{1}{|C|} \sum_{\theta \in C} h_\theta(x) \tag{25}$$

- $d(x)$ represents the diversity measure, computed as the minimum distance to previously selected instances in the query batch:

$$d(x) = \min_{x' \in Q} \text{dist}(x, x') \tag{26}$$

where $\text{dist}(x, x')$ denotes a specific distance metric (e.g., Euclidean distance, cosine similarity, or Random Forest dissimilarity).
- $h(x)$ represents the density measure, calculated using the k-nearest neighbors (k-NN) approach:

$$h(x) = \frac{1}{k} \sum_{x' \in N_k(x)} \text{sim}(x, x') \tag{27}$$

where $N_k(x)$ denotes the k-nearest neighbors of x, and $\text{sim}(x, x')$ represents the similarity between instances.

A0.7 GSx/GSy/iGS

GSx (Greedy Sampling in the Input Space), GSy (Greedy Sampling in the Output Space), and iGS (Improved Greedy Sampling in both Input and Output Spaces) are AL for regression methods based on Greedy Sampling (GS), proposed by Wu *et al.*

1. GSx: Greedy Sampling in the Input Space

GSx primarily focuses on the diversity of the input feature space to ensure that selected samples are evenly distributed in the feature space. The core steps are:

- **Initialization**: Select the first sample closest to the centroid of all unlabeled samples.
- **Iterative selection**: For each unlabeled sample x_n, compute its minimum distance to all labeled samples:

$$d_{x_n} = \min_{x_m \in S} ||x_n - x_m|| \tag{28}$$

- **Selection**: Choose the sample with the maximum d_{x_n} for labeling to maximize coverage in the input space.

GSx has low computational cost and does not depend on the predictions of the regression model, making it suitable as a passive sampling strategy.

2. GSy: Greedy Sampling in the Output Space

GSy aims to maximize diversity in the output space to enhance model prediction capability. Since output labels are unknown in the unlabeled set, GSy requires at least K_0 labeled samples to train an initial regression model $f(x)$ before applying output-space-based greedy sampling. The steps are:

- **Initialization**: Select the first K_0 samples using GSx for labeling and train an initial regression model.
- **Iterative selection**: For each unlabeled sample x_n, compute the minimum distance between its predicted output and labeled samples:

$$d_{y_n} = \min_{y_m \in S} ||f(x_n) - y_m|| \tag{29}$$

- **Selection**: Choose the sample with the maximum d_{y_n} for labeling to maximize coverage in the output space.

GSy updates the regression model after each sampling step, making it computationally more expensive but significantly improving regression accuracy.

3. iGS: Improved Greedy Sampling in Input and Output Spaces

iGS integrates both GSx and GSy strategies, considering diversity in both the input and output spaces to maximize information gain from new samples. The core steps are:

- **Initialization**: Select the first K_0 samples using GSx and train the initial regression model.
- **Iterative selection**: For each unlabeled sample x_n, compute its minimum distances in both the input and output spaces:

$$d_{x_n} = \min_{x_m \in S} ||x_n - x_m||, \quad d_{y_n} = \min_{y_m \in S} ||f(x_n) - y_m|| \tag{30}$$

- **Joint selection criterion**: Compute the final selection score as:

$$d_{xy_n} = \min(d_{x_n}, d_{y_n}) \tag{31}$$

- **Selection**: Choose the sample with the maximum d_{xy_n} for labeling to ensure balanced coverage in both input and output spaces.

iGS ensures that while maintaining diversity in the input space, the model also improves its predictive capability for unlabeled data.

A0.8 RD-ALR

Wu *et al.* [8] proposed four pool-based sequential AL for regression (ALR) algorithms: **RD (Representativeness and Diversity Sampling)**, **RD-QBC (RD integrated with Query-by-Committee)**, **RD-EMCM (RD integrated with Expected Model Change Maximization)**, and **RD-GS (RD integrated with Greedy Sampling)**. The four RD-ALR algorithm variants and their principles are shown in Tab. 6.

RD applies an *unsupervised clustering approach* for initial sample selection and ensures diversity in subsequent iterations. The first labeled samples are selected using **k-means clustering**, with cluster centroids chosen for labeling to ensure representativeness. In sequential sampling, RD re-applies **k-means clustering with k = m** (where m is the number of labeled samples) and selects the unlabeled sample closest to the centroid of the largest cluster.

RD-QBC extends RD by incorporating **Query-by-Committee (QBC)** to account for informativeness. Instead of directly selecting the cluster centroid, RD-QBC selects the most informative sample within the cluster based on committee disagreement. The uncertainty is measured by the variance of predictions across committee members:

$$\sigma_n = \frac{1}{P}\sum_{p=1}^{P}(y_{pn} - \bar{y}_n)^2 \tag{32}$$

where y_{pn} is the prediction of the p-th model, and $\bar{y}_n$ is the mean prediction.

RD-EMCM incorporates **Expected Model Change Maximization (EMCM)** into RD for informativeness-driven sampling. Instead of selecting the cluster centroid, RD-EMCM selects the sample expected to cause the largest model update. The informativeness score is defined as:

$$g(x_n) = \frac{1}{P}\sum_{p=1}^{P}||(y_{pn} - \hat{y}_n)x_n|| \tag{33}$$

where $\hat{y}_n$ is the model's current prediction for sample x_n.

RD-GS enhances **Greedy Sampling (GS)** by integrating RD's clustering-based representativeness. Instead of selecting the cluster centroid, RD-GS prioritizes the sample farthest from previously labeled points:

$$d_n = \min_{x_m \in S}||x_n - x_m|| \tag{34}$$

where S is the set of labeled samples.

Algorithm	Core Strategy
RD	Representativeness + Diversity (Clustering-based)
RD-QBC	Representativeness + Diversity + QBC (Variance-based)
RD-EMCM	Representativeness + Diversity + EMCM (Model Change)
RD-GS	Representativeness + Diversity + Greedy Sampling (Distance-based)

Table 6. Four variants of RD-ALR, each combining representative sampling with diversity sampling and introducing an additional information component.

RD ensures balanced sample distribution, suitable for regression tasks with unknown data distributions. RD-QBC and RD-EMCM prioritize informative samples, improving model learning efficiency. RD-GS emphasizes spatial diversity, making it effective in high-dimensional datasets. Experimental results on **11 regression datasets** demonstrate that the RD series outperforms traditional QBC, EMCM, and GS methods, achieving superior data efficiency and generalization capability in AL scenarios.

A0.9 mcdropout (MCDO)

This study also evaluates the Monte Carlo Dropout (MC-Dropout) based AL method implemented in the **BaaL framework**, developed by Atighehchian *et al.* [9]. This method leveraged MC-Dropout to estimate **epistemic uncertainty**, enabling the model to actively query the most uncertain samples for labeling. Specifically, this approach employs **DL-based uncertainty estimation** and utilizes **Monte Carlo Sampling** to obtain predictive distributions for each unlabeled sample, thereby quantifying model uncertainty.

In implementation, a **multi-layer perceptron (MLP) neural network** is constructed. The network structure is shown in **??**. The trained model then utilizes **MCDropoutConnectModule** during inference, maintaining the dropout mechanism during the prediction phase. Once trained, the model performs T **Monte Carlo forward passes** over the **unlabeled pool** D_U in each AL

iteration, computing predictive means and standard deviations. Finally, the samples with the highest predictive variance are selected for labeling to optimize data utilization and enhance model generalization.

Formally, given the unlabeled dataset D_U and the trained neural network $f(x,w)$, MC-Dropout estimates the uncertainty by performing T **stochastic forward passes**:

$$p_t(y|x) = f(x, w_t), \quad w_t \sim p(w|D) \tag{35}$$

where T represents the number of Monte Carlo samples. The predictive variance is then computed as:

$$\sigma^2(x) = \frac{1}{T}\sum_{t=1}^{T}(p_t(y|x) - \bar{p}(y|x))^2 \tag{36}$$

where $p_t(y|x)$ is the prediction from the t-th forward pass, and $\bar{p}(y|x)$ is the mean prediction across all forward passes. Samples with higher variance are considered the most uncertain and are thus selected for labeling.

A0.10 LL4AL

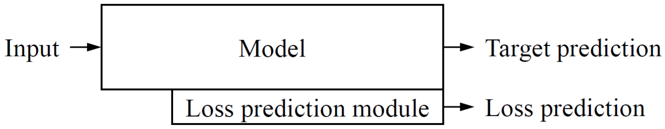

(a) A model with a loss prediction module

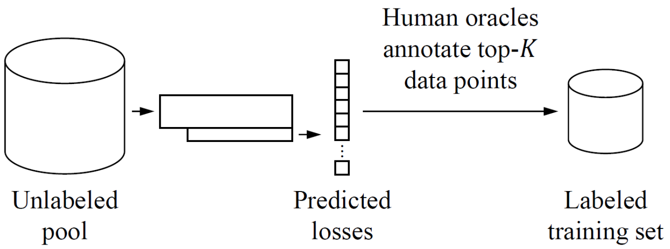

(b) Active learning with a loss prediction module

Figure 7. LL4AL AL method. (a) A loss prediction module attached to a target model predicts the loss value from an input without its label. (b) All data points in an unlabeled pool are evaluated by the loss prediction module. The data points with the top-K predicted losses are labeled and added to a labeled training set. [10].

Neural networks learn by minimizing loss. In each iteration, the network calculates the difference between the predicted and actual values, updating its parameters to make the predictions more accurate. Loss measures the model's prediction ability: a smaller loss indicates good performance, whereas a larger loss suggests the model struggles with certain samples, making them crucial for improving the model. Thus, if the loss for all samples can be determined, it can be used to assess the importance of each sample—the larger the loss, the more important the sample.

However, for datasets where only some data points are labeled, the loss for unlabeled data cannot be directly computed. Yoo *et al.* [10] developed LL4AL (Loss Learning for AL), which provides a method to predict possible losses for unlabeled data by leveraging the features of unlabeled samples and the hidden layers of the neural network. Fig. 7 describes the working principle of LL4AL. LL4AL is a model-based AL strategy. It integrates a small neural network, called the **loss prediction module**, into the main neural network. This module is designed to predict the loss of unlabeled data and assist in sample selection. The main network performs the usual predictive tasks, while the loss prediction module continuously learns and updates during each AL cycle. Eventually, it can estimate the loss of unlabeled samples and guide the selection of the next batch of samples.

As a DL-based AL strategy, the neural network structure used for fitting the data is consistent with the method **mcdropout**, as shown in **??**.

A0.11 LCMD

This study evaluates **LCMD**, a method proposed by Holzmüller *et al.* [11], known as **Largest Cluster Maximum Distance (LCMD)**.This approach is designed for **Deep Batch Active Learning (DBAL)** tasks, combining **data clustering** and

maximum distance sampling to ensure that selected samples are both **informative (high uncertainty)** and **representative (covering high-density regions)**.

LCMD first **clusters the unlabeled data** and then selects samples using a **greedy maximum distance strategy** within the **largest cluster**, ensuring an optimal balance between diversity and learning efficiency.The specific steps are:

1. Largest Cluster Selection:

- Apply **k-means** clustering to partition the unlabeled dataset into clusters.
- Identify the **largest cluster** , which contains the highest number of samples.

2. Maximum Distance Sampling:

- Select the most uncertain sample from the largest cluster:

$$x_1 = \arg\max_{x \in C_{\max}} k(x,x) \tag{37}$$

- Iteratively select samples that are maximally distant from the previously chosen set , ensuring diversity:

$$x_t = \arg\max_{x \in C_{\max}} \min_{x' \in S_t} d_k(x,x') \tag{38}$$

- Repeat until the batch size is reached.

As a DL-based AL strategy, the neural network structure used for fitting the data is consistent with the method **mcdropout**, as shown in **??**.

Appendix B

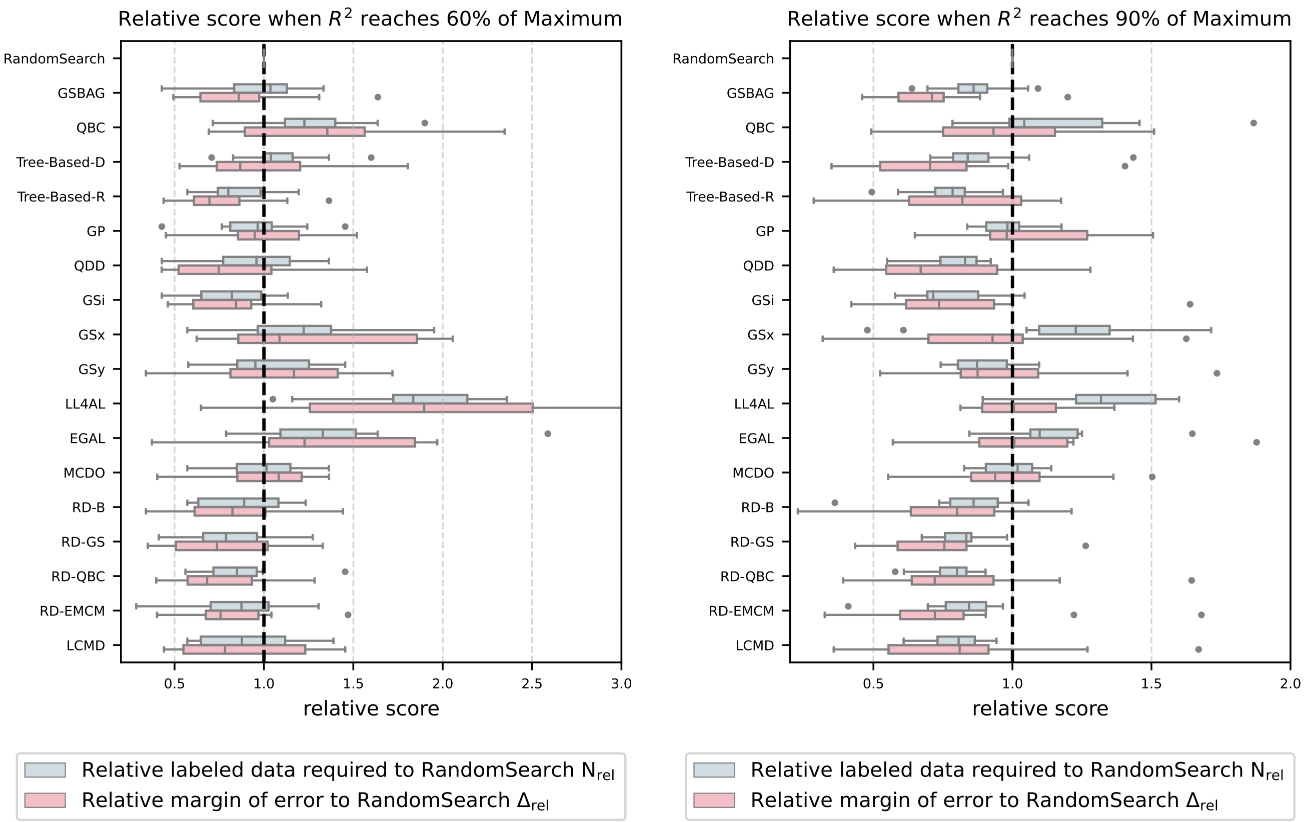

Figure 8. Distribution of the relative score (N_{rel}, Δ_{rel}) of each AL strategy when reaching 60% and 90% of the maximum R^2 score

Appendix C

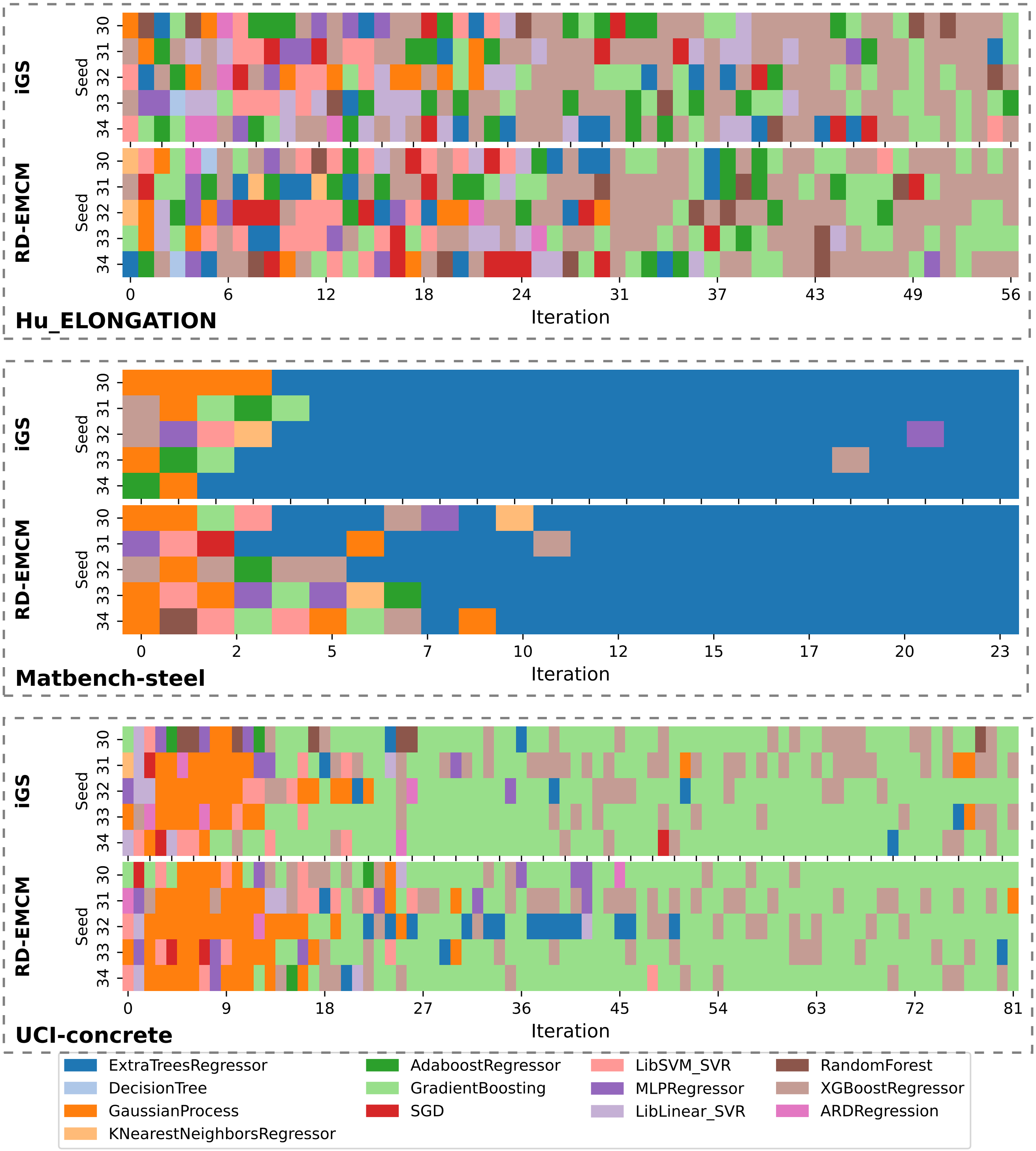

Figure 9. Heatmaps showing the dominant model selected by AutoML ensembles after each active learning iteration with two strategies (iGS and RD-EMCM) on three benchmark datasets (Hu_ELONGATION, Matbench-steel, UCI-concrete). Colors indicate different model types

Appendix D

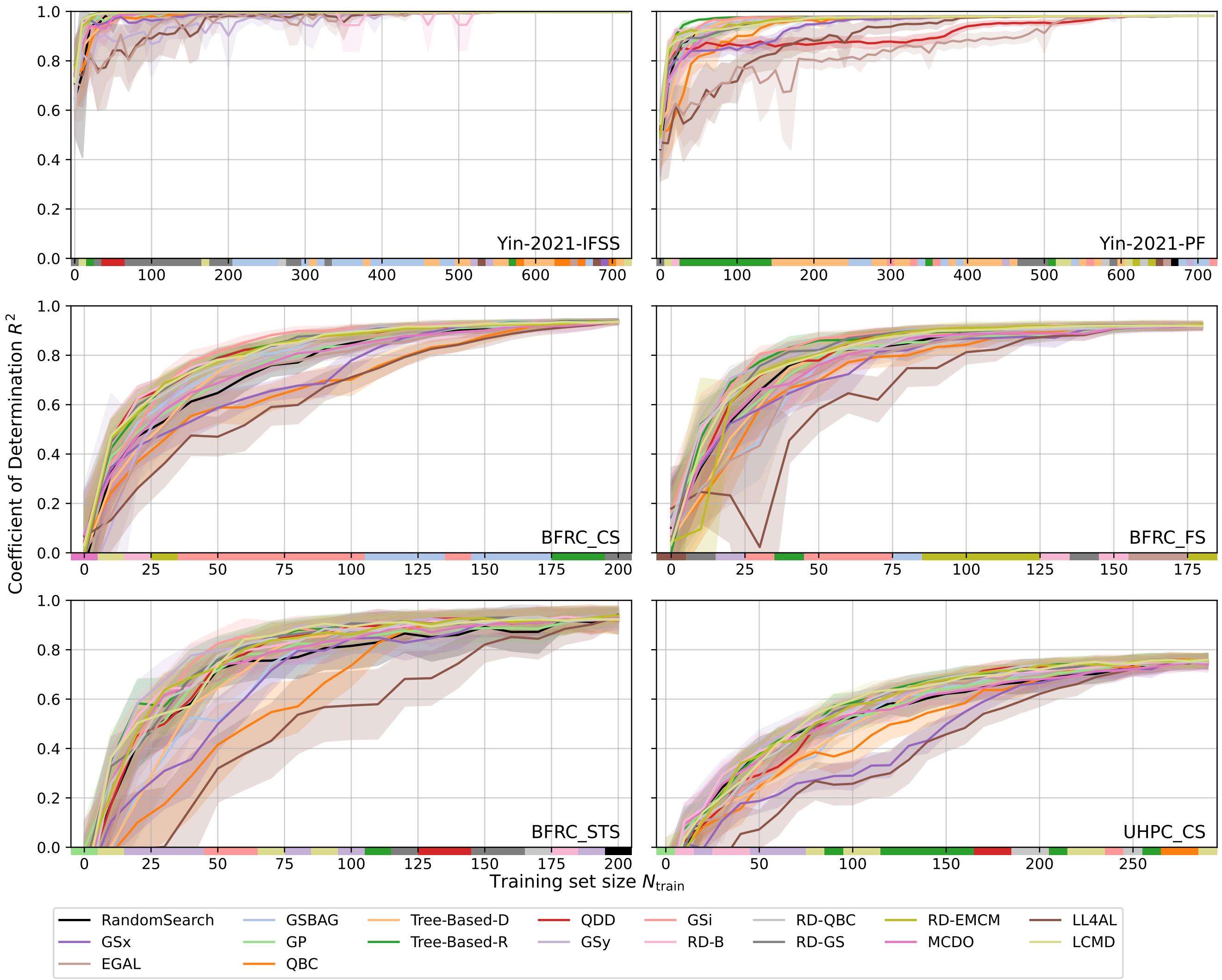

Figure 10. R^2 score trends for all tested AL strategies on selected datasets. The color bar shows the colors represented by the AL strategies that had the best model performance (maximum R^2 score) for a given training set sample size.

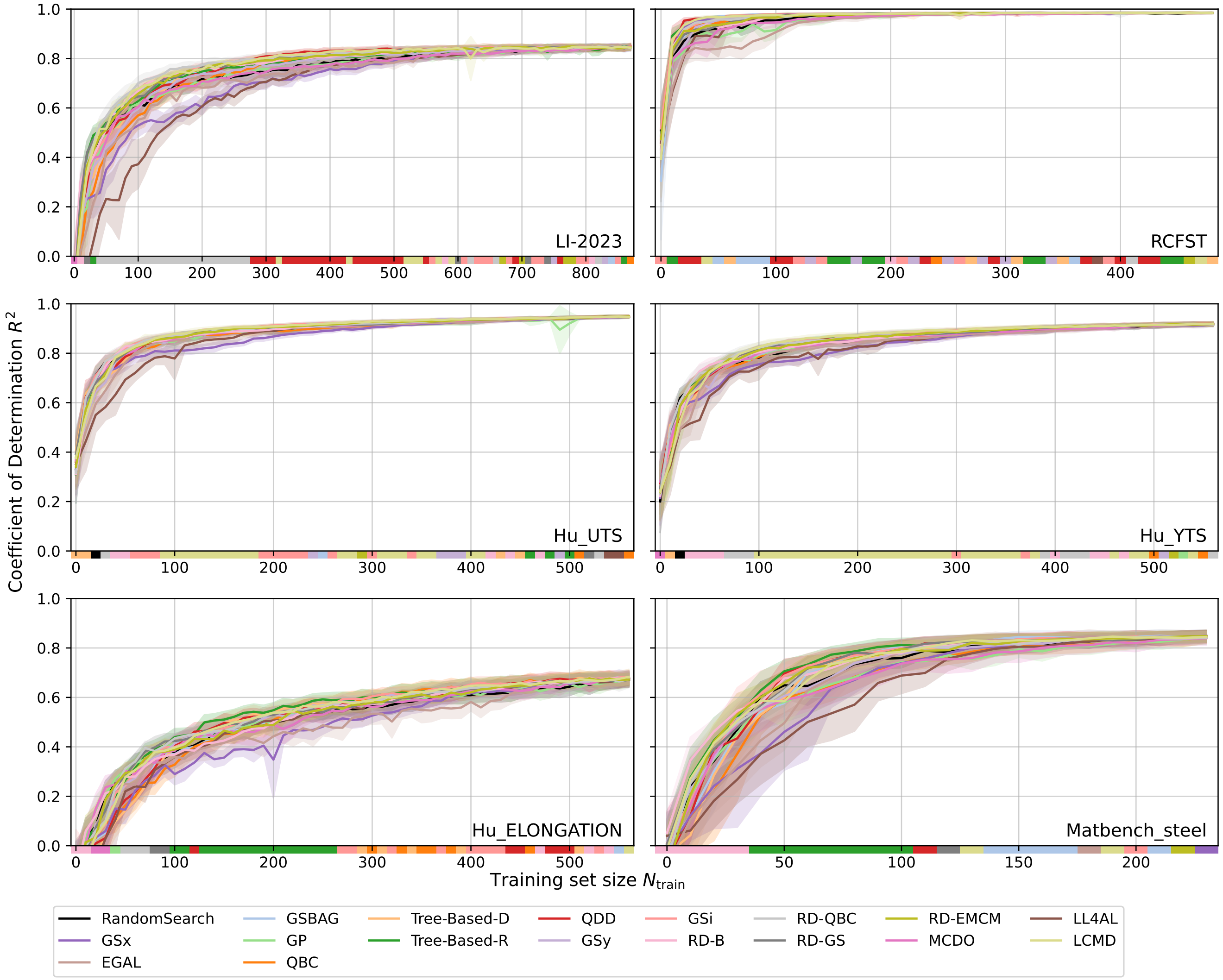

Figure 11. R^2 score trends for all tested AL strategies on selected datasets. The color bar shows the colors represented by the AL strategies that had the best model performance (maximum R^2 score) for a given training set sample size.

Appendix E

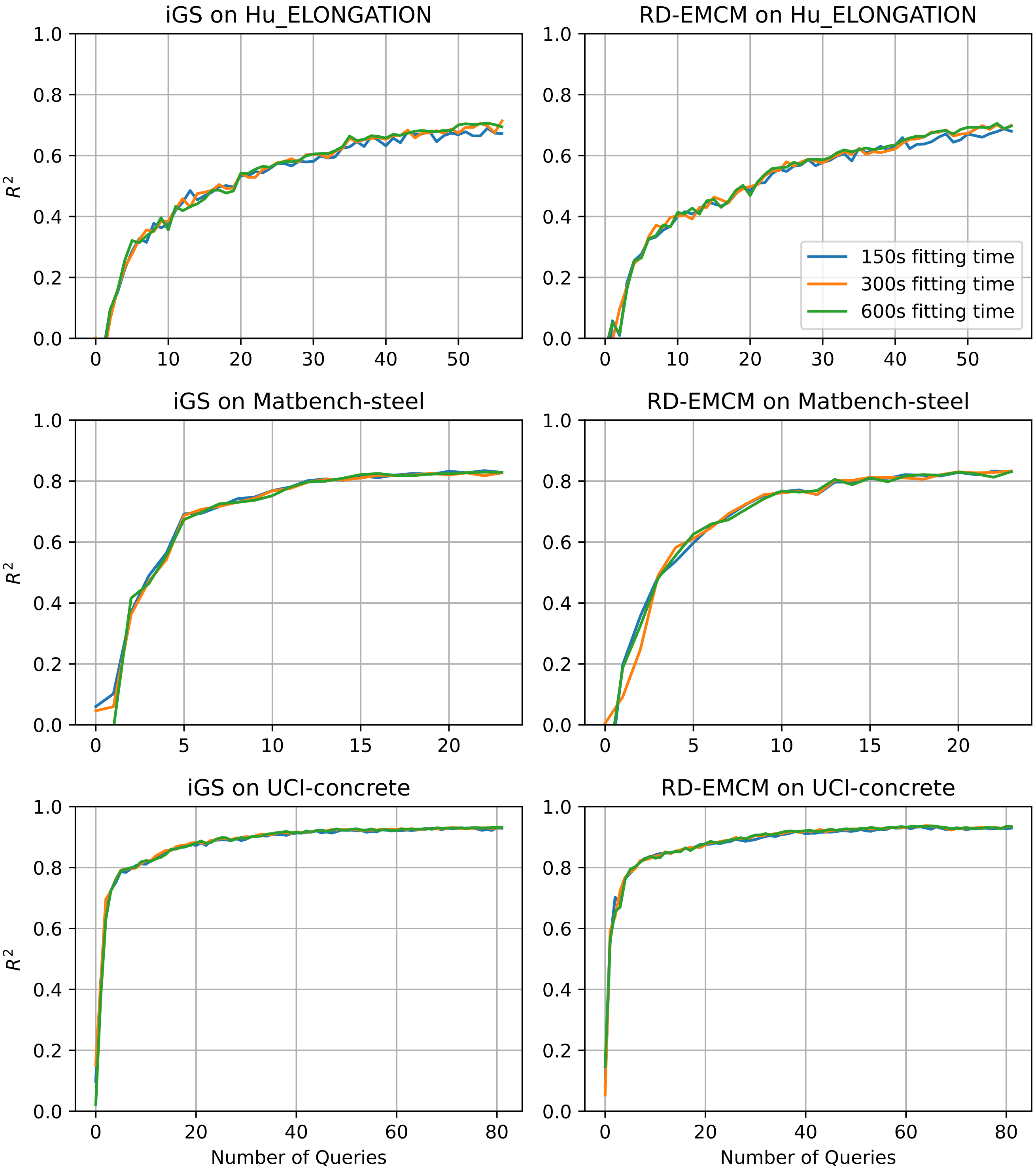

Figure 12. R^2 learning curves of two active learning strategies (iGS and RD-EMCM) on three datasets (Hu-2021(ELONGATION), Matbench-steel and UCI-concrete) under different AutoML fitting time limits (150s, 300s, 600s).

Appendix F

Strategy	Max time per acquisition (s)	Relative cost
TreeBased-Representativity	0.47	Negligible
GaussianProcessBased	0.0008	Negligible
TreeBased-Diversity	0.02	Negligible
EGAL	0.11	Negligible
TreeBased-Representativity	0.47	Negligible
Basic_RD_ALR	1.50	Low
RD_GS_ALR	1.74	Low
GSBAG	1.97	Low
mcdropout	3.85	Low
BMDAL	7.57	Moderate
RD_EMCM_ALR	12.11	Moderate
LearningLoss	12.27	Moderate
GSx	16.15	Moderate
QueryByCommittee	21.39	Moderate
GSy	26.80	Moderate
QDD	63.82	High
GSi	70.33	High
RD_QBC_ALR	233.97	Very High

Table 7. Maximum computing time per acquisition step for all AL strategies across three representative datasets

References

1. Hu, R., Jane Delany, S. & Mac Namee, B. Egal: Exploration guided active learning for tcbr. In *International Conference on Case-Based Reasoning*, 156–170 (Springer, 2010).

2. Zhao, Y., Lin, J., Lin, J. & Wu, E. Q. Batch-mode active learning of gaussian process regression with maximum model change. *IEEE Transactions on Syst. Man, Cybern. Syst.* (2023).

3. Pasolli, E. & Melgani, F. Gaussian process regression within an active learning scheme. In *2011 IEEE International Geoscience and Remote Sensing Symposium*, 3574–3577 (IEEE, 2011).

4. Paleyes, A., Mahsereci, M. & Lawrence, N. D. Emukit: A python toolkit for decision making under uncertainty. In *Python in Science Conference*, 68–75 (2023).

5. Burbidge, R., Rowland, J. J. & King, R. D. Active learning for regression based on query by committee. In *Intelligent Data Engineering and Automated Learning-IDEAL 2007: 8th International Conference, Birmingham, UK, December 16-19, 2007. Proceedings 8*, 209–218 (Springer, 2007).

6. Jose, A. *et al.* Regression tree-based active learning. *Data Min. Knowl. Discov.* **38**, 420–460 (2024).

7. Kee, S., Del Castillo, E. & Runger, G. Query-by-committee improvement with diversity and density in batch active learning. *Inf. Sci.* **454**, 401–418 (2018).

8. Wu, D. Pool-based sequential active learning for regression. *IEEE transactions on neural networks learning systems* **30**, 1348–1359 (2018).

9. Atighehchian, P., Branchaud-Charron, F. & Lacoste, A. Bayesian active learning for production, a systematic study and a reusable library. *arXiv preprint arXiv:2006.09916* (2020).

10. Yoo, D. & Kweon, I. S. Learning loss for active learning. In *Proceedings of the IEEE/CVF conference on computer vision and pattern recognition*, 93–102 (2019).

11. Holzmüller, D., Zaverkin, V., Kästner, J. & Steinwart, I. A framework and benchmark for deep batch active learning for regression. *J. Mach. Learn. Res.* **24**, 1–81 (2023).
